# Autophagy modulators influence the content of important signalling molecules in PS-positive extracellular vesicles

**DOI:** 10.1186/s12964-023-01126-z

**Published:** 2023-05-24

**Authors:** Klara Hanelova, Martina Raudenska, Monika Kratochvilova, Jiri Navratil, Tomas Vicar, Maria Bugajova, Jaromir Gumulec, Michal Masarik, Jan Balvan

**Affiliations:** 1 Department of Pathological Physiology, Faculty of Medicine, Masaryk University, Kamenice 5, 625 00 Brno, Czech Republic; 2 Department of Physiology, Faculty of Medicine, Masaryk University, Kamenice 5, 625 00 Brno, Czech Republic; 3 Department of Biomedical Engineering, Faculty of Electrical Engineering and Communication, Brno University of Technology, Technicka 3058/10, Brno, Czech Republic; 4 First Faculty of Medicine, Charles University, Katerinska 32, 12108 Prague, Czech Republic

**Keywords:** Extracellular vesicles, Phosphatidylserine-positive extracellular vesicles, Autophagy, Autophagy modulation, Head and neck cancer, Proteomics, SQSTM1, Senescence, p21, Autophinib, CPD18, EACC, Bafilomycin A1, 3-hydroxychloroquine, Rapamycin, NVP-BEZ235, Torin1, Starvation

## Abstract

Extracellular vesicles (EVs) are important mediators of intercellular communication in the tumour microenvironment. Many studies suggest that cancer cells release higher amounts of EVs exposing phosphatidylserine (PS) at the surface. There are lots of interconnections between EVs biogenesis and autophagy machinery. Modulation of autophagy can probably affect not only the quantity of EVs but also their content, which can deeply influence the resulting pro-tumourigenic or anticancer effect of autophagy modulators. In this study, we found that autophagy modulators autophinib, CPD18, EACC, bafilomycin A1 (BAFA1), 3-hydroxychloroquine (HCQ), rapamycin, NVP-BEZ235, Torin1, and starvation significantly alter the composition of the protein content of phosphatidylserine-positive EVs (PS-EVs) produced by cancer cells. The greatest impact had HCQ, BAFA1, CPD18, and starvation. The most abundant proteins in PS-EVs were proteins typical for extracellular exosomes, cytosol, cytoplasm, and cell surface involved in cell adhesion and angiogenesis. PS-EVs protein content involved mitochondrial proteins and signalling molecules such as SQSTM1 and TGFβ1 pro-protein. Interestingly, PS-EVs contained no commonly determined cytokines, such as IL-6, IL-8, GRO-α, MCP-1, RANTES, and GM-CSF, which indicates that secretion of these cytokines is not predominantly mediated through PS-EVs. Nevertheless, the altered protein content of PS-EVs can still participate in the modulation of the fibroblast metabolism and phenotype as p21 was accumulated in fibroblasts influenced by EVs derived from CPD18-treated FaDu cells. The altered protein content of PS-EVs (data are available via ProteomeXchange with identifier PXD037164) also provides information about the cellular compartments and processes that are affected by the applied autophagy modulators.

Video Abstract

**Supplementary Information:**

The online version contains supplementary material available at 10.1186/s12964-023-01126-z.

## Introduction

The evolutionary success of tumour cells largely depends on the manipulation of their tumour microenvironment (TME), especially cancer-associated fibroblasts (CAFs) [1, 2]. Fibroblasts reflect the stress within the TME in a variety of ways that allow them to adapt to stressful conditions. Nevertheless, such adaptation can enhance the malignant behaviour of tumour cells as stress responses in fibroblasts include their activation to CAFs or senescence [3]. Key mediators of intercellular communication are extracellular vesicles (EVs). While originally thought to function mainly in the elimination of cellular waste, it is now understood that the purpose of EVs is much more complex [4]. EVs involve a heterogeneous population of membrane vesicles that are created through various mechanisms. The three main EV subpopulations include bodies released by apoptotic cells (1,000–5,000 nm), ectosomes (150–1,000 nm), and exosomes (30–150 nm). Apoptotic bodies are released from dying cells and are usually pelleted at 2000 to 10,000 g. Ectosomes comprise diverse types of EVs such as oncosomes and microvesicles that are generated at the plasma membrane from its outward budding and are isolated at 10,000 to 20,000 g [5, 6].

Exosomes are a subtype of EVs generated by an endosomal system, pelleted at > 100,000 g centrifugation [6, 7]. The release of exosomes involves the formation of multivesicular endosomes (MVEs) that, upon transport towards and fusion with the plasma membrane, release their intraluminal vesicles (ILVs) as exosomes [4]. Exosome biogenesis is complex, varies depending on the cargo and the cell type they are derived from, and can be influenced by other signals and pathological stimuli. EVs mirror the composition of their parent cells and contain various biomolecules, including proteins, lipids, and nucleic acids, which can influence the behaviour of specific target cells. Some studies suggest that cancer cells release higher amounts of extracellular vesicles and that EVs produced by tumour cells often expose phosphatidylserine (PS) at the surface [8–10].

Autophagic machinery collectively with the ubiquitin–proteasome system and exosomal secretion orchestrate the dynamics of intracellular waste removal, where each pathway may complement the deficit of the other. Consequently, the secretion of EVs with waste material (proteins, RNA, parts of organelles) can reduce stress when degradative and recycling pathways are disrupted [11, 12]. Recent studies confirm that there are many interconnections between exosome biogenesis and autophagy and that autophagy machinery can have both stimulatory and inhibitory impacts on the secretion of EVs. These impacts will be probably deeply context-dependent and could be significantly affected by different kinds of autophagy modulators (the significant effect can be expected especially for autophagy modulators impairing lysosome homeostasis [13]). Modulation of autophagy can significantly affect not only the quantity of EVs but also their content, which can deeply influence the resulting pro-tumourigenic or anticancer effect of autophagy modulators [14–17].

In this study, we investigated the impact of autophagy modulators on the protein content of PS-positive EVs (PS-EVs) produced by head and neck cancer cells (HNSCC) and following communication between cancer cells and fibroblasts. The effect of HNSCC cells-conditioned media containing PS-EVs (derived from cancer cells with and without autophagy modulation) on the metabolism and senescence-associated proteins of healthy fibroblasts was studied. Nine modulators were chosen for modulation of the autophagy machinery (autophinib (APB), CPD18, EACC, bafilomycin A1 (BAFA1), 3-hydroxychloroquine (HCQ), rapamycin (RAPA), NVP-BEZ235 (BEZ), Torin1, and starvation). Autophinib is an ATP-competitive inhibitor of VPS34 decreasing the accumulation of the lipidated protein LC3 on the autophagosomal membrane. CPD18 inhibits omegasome formation via the inhibition of class III PI3K. CPD18 is structurally related to 3-methyladenine (3-MA), but unlike 3-MA, CPD18 does not inhibit class I PI3Ks [18]. EACC is a reversible autophagy inhibitor, which selectively inhibits the translocation of autophagosome-specific SNARE Stx17, thereby blocking autophagosome-lysosome fusion. EACC blocks autophagosome-lysosome fusion but does not affect endo-lysosomal function [19]. BAFA1 is a V-ATPase inhibitor that blocks the autophagic flux by inhibiting autophagosome-lysosome fusion and autolysosomal and/or lysosomal acidification [20]. HCQ inhibits the maturation of autolysosomes and blocks late steps of autophagy. HCQ behaves as a weak base and accumulates in the lysosomes causing lysosomal stress [21]. Rapamycin is a potent allosteric mTORC1 inhibitor. NVP-BEZ235 binds to the ATP-binding clefts of PI3K and mTOR kinase, thereby inhibiting their activities [22]. Torin1 is a highly potent and selective ATP-competitive mTOR inhibitor that directly inhibits both complexes (mTORC1 and mTORC2) [23].

We found that autophagy modulators significantly altered the composition of the protein content of PS-positive EVs (PS-EVs) produced by cancer cells. This protein content involves important cytokines, mitochondrial proteins, and signalling molecules such as SQSTM1 and TGFβ1 pro-protein. The altered protein content of PS-EVs may contribute to the modulation of the fibroblast metabolism and phenotype (p21 was accumulated in fibroblasts influenced by EVs derived from CPD18-treated FaDu cells). EVs also provide information about the cellular compartments and processes that are affected by the applied compounds.

## Material and methods

### Model cell lines

The cell line FaDu (HTB-43TM), derived from a squamous cell carcinoma (SCC) of the hypopharynx, and the human gingival fibroblast cell line HGF (derived from the histologically normal gingival biopsy; Cell Lines Service product number: 300703) were used in this study. The authenticated cell lines were purchased from the American Type Culture Collection (ATCC; Manassas, Virginia, USA) and CLS within the last five years. Homozygous deletion of SMAD4 was observed in FaDu cells [24, 25]. SMAD4 gene depletion can induce autophagy. The protein level of SMAD4 was inversely correlated with autophagy in orthotopic tumour tissue samples [26].

The cell lines were grown in a Dulbecco’s Modified Eagle’s medium/Nutrient Mixture F-12 Ham (DMEM/F12, Biosera) supplemented with antibiotics (pen-strep) and 10% fetal bovine serum in a humidified atmosphere of 5% CO_2_ and 95% air at 37 °C. Two passages before the experiment, cell harvest, or EVs isolation, the cell lines were washed with PBS and grown in a medium supplemented with Exosome-depleted FBS (GibcoTM, A2720801); hereafter referred to as exofree medium. The passages of all cell lines performed in our lab ranged from 5 to 15. All experiments were performed with mycoplasma-free cells. Mycoplasma was detected by PCR (primers MYCO_A: GGCGAATGGGTGAGTAACACG and MYCO_B: CGGATAACGCTTGCGACCTATG).

### Autophagy modulation

For autophagy modulation, FaDu cells were treated for 24 h with 5 nM bafilomycin A1 (Sigma-Aldrich, B1793), 50 µM of hydroxychloroquine sulphate (Sigma-Aldrich, H0915), 100 µM of CPD18 (Calbiochem), 50 nM of autophinib (Sigma, SML2632), 10 µM of EACC (MedChemExpress), 200 nM of rapamycin (Sigma-Aldrich, R0395), 3 nM of Torin-1 (MedChemExpress), or 30 nM of NVP-BEZ235 (MedChemExpress). To induce starvation, cells were cultured in DMEM F12 without glutamine and without FBS (Biosera). Modulation of autophagy did not reduce the viability of FaDu cells (the viability ranged 98.1 to 99.9% across treatments).

### Conditioned media preparation

15 ml of conditioned medium (CM) was collected from 90*–*95% confluent FaDu cells grown in 75cm^2^ cell culture flasks after 24 h of the selected treatment. The collected media were first subjected to a centrifugation step of 400 g for 10 min at room temperature (RT) to pellet and remove cells. All following centrifugation steps were performed at 4 °C. Next, the supernatant was centrifuged at 2,000 g for 20 min to remove debris and apoptotic bodies [6]. Then, to remove large EVs, the supernatant was centrifuged at 10,000 g for 40 min. To remove any remaining large EVs, the supernatant from the first 10,000 g step was passed through a 0.22 µm pore PES filter (MF-Millipore TM Membrane Filter, GSWP01300). The conditioned medium resulting from this process is hereinafter referred to as CM1.

To remove the remaining exogenous treatments from the CM1, we concentrated the 15 ml volume to a 1 ml volume using Amicon® Ultra-15 Centrifugal Filter Unit (Merc, UCF9100, 100 kDa MWCO) at 2000 g for 3 min. Then we added 2 ml of pure DMEM F12 medium supplemented with exosome-depleted FBS to this volume. The conditioned medium resulting from this process is hereinafter referred to as CM2. This conditioned medium was subsequently used for the treatment of HGF cells.

### Cytokine array

Human Cytokine Antibody Arrays (ab 133,996 and ab133997, Abcam) were used for the simultaneous detection of 23 resp. 42 cytokines in each CM sample according to the manufacturer’s instructions. The array membranes were incubated for 30 min at room temperature in a blocking buffer. CM samples were then incubated on the membranes overnight at 4 °C. Following one large volume wash in Wash buffer I for an extended time of 30 min, three washes in Wash buffer I and two washes in Wash buffer II, membranes were incubated in Biotin-Conjugated Anti-Cytokines overnight at 4 °C. After washing as described above, membranes were incubated in HRP-Conjugated Streptavidin overnight at 4 °C. Washed arrays were finally incubated with Chemiluminescence Detection reagents and images were captured using the Azure c400 Imager (Azure Biosystems). Pixel density (signal density) of each spot on the membrane was quantified using ImageJ—Array Analysis plugin [27].

### Extracellular vesicle isolation and characterization

For all EVs-isolation methods, CM2 was used. During processing, the conditioned medium was kept on ice. EVs were isolated from the pre-cleared concentrated medium (CM2) according to the manufacturer’s instructions. The following kits were used for EVs isolation by immunoaffinity methods: (Exosome-Human EpCAM Isolation Reagent, Invitrogen; Exosome Isolation Kit CD63, Miltenyi Biotec; MagCapture™ Exosome Isolation Kit PS, FUJIFILM Wako Pure Chemical Corporation, Japan). For ultracentrifugation, the CM2 was transferred to a fresh 38 ml thin-wall polyallomer SW 28 UC tube and centrifuged for 120,000 g for 4 h in an SW 28 Ti Swinging Bucket rotor (k-factor of 246) using Optima XPN 90 K Ultracentrifuge (Beckman Coulter). The crude EVs pellet was resuspended in a large volume of PBS followed by ultracentrifugation at 120,000 g for 4 h to wash the sample. The pellet was placed in filtered ice-cold PBS. All isolated EVs were then placed in 1,5 ml Protein LoBind Tubes (Sigma, EP0030108116) and stored in the manufacturers’ elution buffers (filtered PBS in the case of ultracentrifugation) at -80 °C.

According to the MISEV2018 recommendations [28], five categories of proteins should be evaluated when characterizing EVs. The first category includes transmembrane proteins or glycosylphosphatidylinositol (GPI)-anchored proteins localized in the plasma membrane or endosomes, the presence of which demonstrates a lipid bilayer specific for EVs. The second category includes cytosolic proteins enclosed in EVs. The third category includes proteins often isolated together with EVs (e.g., proteins contained in the cytoplasm outside EVs, culture medium, serum, or urine). Evaluation of their presence helps to assess the degree of purity of isolated EVs. The fourth category includes transmembrane, lipid-bound, and soluble proteins associated with intracellular compartments other than the plasma membrane/endosomes (i.e., the membrane donor may be mitochondria, endoplasmic reticulum, Golgi apparatus, autophagosomes). The fifth category includes secreted or luminal proteins that may associate with EVs through binding to receptors on the surface of EVs; often cytokines, interleukins, and growth factors, which may represent a functional component of EVs in intercellular communication. The fifth category of proteins also reflects the native state of isolated exosomes (ligand-receptor binding is preserved). After evaluating all protein categories by LC–MS, the phosphatidylserine (PS)-based MagCapture™ method was judged to be the best method for isolating native EVs; see Additional file 1 Fig. S1. Due to the high viability of the observed cell populations after exposure to individual compounds and due to the size of the observed EVs, contamination of PS-EVs with apoptotic bodies is not expected. Moreover, apoptotic bodies are usually pelleted at low centrifugation, such as 2000 to 10,000 g [6]. Ribosomal proteins (RPS27A, RPS8) were found to be the largest contaminants of isolated EVs.

### Dynamic Light Scattering (DLS)

Prior to the DLS measurements, each EVs sample (PS-positive EVs) was gently shaken at 4 °C for 20 min to dissolve possible EVs aggregates. About 50µL of each sample was added to a low-volume quartz batch cuvette (ZEN2112, Malvern Panalytical). DLS measurements were conducted at 25 °C using a Zetasizer Ultra (Malvern Instruments Ltd., UK) operating at 633 nm and recording the back scattered light at an angle of 173°. The sample temperature was allowed to equilibrate for 10 min before each measurement. The light scattering was recorded for 2 s with 35 replicate measurements (35 runs). The data were processed using the ZS Xplorer software (Malvern Panalytical).

### Negative stain transmission electron microscopy and cryo-electron microscopy

EVs fractions (protein concentrations ranging from 243 to 417 µg/ml) were prepared for transmission electron microscopy (TEM) by absorption at activated Formvar/Carbon coated grids (HF35Cu, Pyser-SGI Limited, Kent, UK) for 5 min at RT. This was accomplished by pipetting 10 µl of the EVs containing elution buffer on the grid. Following sample adsorption, grids were quickly and gently blotted on filter paper and immediately floated for 5 min on 1 mL of 2% uranyl acetate and dried on filter paper at RT. EVs were examined at 70 kV with a Morgagni 268D transmission electron microscope (ThermoScientific) equipped with a MegaViewIII digital camera (Soft Imaging System). In all cases, TEM was performed on a fresh sample of EVs that had not been subjected to freezing temperatures at any step of purification or processing.

For cryo-electron microscopy, 3.5 µl of the EVs sample was applied to freshly plasma-cleaned TEM grids (Quantifoil, Cu, 200mesh, R2/1) and vitrified into liquid ethane using ThermoScientific Vitrobot Mark IV (18 °C, 100% rel. humidity, 30 s waiting time, 4 s blotting time). The grids were subsequently mounted into the Autogrid cartridges and loaded to Talos Arctica (ThermoScientific) transmission electron microscope for imaging. The microscope was operated at 200 kV. The EVs cryo-TEM micrographs were collected on Falcon3 direct electron detection camera at the 73,000 × and 120,000 × nominal magnification with the under focus in the range 3–10 µm and the overall dose of < 20 e/Å2.

### Fluorescence microscopy

The autophagosomal/autolysosomal/lysosomal dynamics of affected cells were observed using the combination of PremoTM Autophagy Tandem Sensor (P36239, Invitrogen) with the far-red emitting LysoTracker® Deep Red (L12492, Invitrogen) (Ex 647 nm/Em 668 nm). By combining acid-sensitive Emerald GFP (eGFP) (Ex 488 nm/Em 509 nm) with acid-insensitive TagRFP (Ex 555 nm/Em 584 nm) in the PremoTM kit, autophagosomes and autolysosomes labelling (yellow and red, respectively) is possible.

Immediately after transduction with 12 µl PremoTM Autophagy Tandem Sensor/2 ml cell suspension, cells were seeded at 5 × 10^5^ into 35-mm glass-bottomed gelatin-coated dishes (Ibidi, μ-Dish 35 mm, high Glass Bottom) and cultured for 48 h to equilibrate expression levels. Subsequently, cells were exposed to the selected agents for 6, 12, 24 and 48 h before imaging. LysoTracker® Deep Red staining was performed 1 h before imaging.

To monitor the uptake of isolated PS-EVs by fibroblasts, we stained EVs with PKH67 (Sigma, PKH67GL) and then removed the remaining dye using Exosome Spin Columns (MW 3000) (Thermo Scientific, #4484449). The stained EVs were then suspended in 400 µl of cultivation medium and added to HGF cells growing for 24 h in Ibidi µ-Slide I Luer (Ibidi, 80176). Image acquisition was performed 24 h after EVs addition. 1 µl of 1 µg/ml of Hoechst 33342 (Enzo) (Ex 350 nm/ Em 461 nm) nuclear stain was added 1 h before imaging.

To determine the viability of the cell population prior to isolation of EVs, cells were left in a culture dish with 1 ml of culture medium to which propidium iodide (Sigma-Aldrich) and Hoechst 33342 were added 45 min before capturing. Cells positive for propidium iodide signal were considered dead. Images were automatically processed using custom MATLAB software, where live and dead cells were automatically detected and counted for individual fluorescence channels, which resulted in viability for individual fields of view. Automatic cell detection was based on Laplacian of Gaussian filter (sigma = 6), which was post-processed with maxima detector with required minimal distance between cells (10 px) and intensity threshold.

To maximize the possibility of comparison between samples, all samples (from a single cell line) were captured on the same day in a single run using the same microscope settings. For each time and each treatment, 10–12 fields of view were captured from randomized sites of the culture dish. Confocal microscopy images were acquired using Laser scanning confocal microscope Zeiss LSM 880 with AiryscanFast module (Carl Zeiss Inc.) using a C-Apochromat 40x/1.20W and C-Apochromat 63x /1.20W. LysoTracker® Deep Red was excited with 633 nm solid-state laser and emitted light was detected at 638–759 nm. Emerald GFP was excited with 488 nm using Argon laser, and emitted light was detected at 493–576 nm. TagRFP and PI were excited using DPSS 561 nm laser, and emitted light was detected at 570–650 nm. Hoechst 33342 was excited with a 405 nm solid-state laser, and emitted light was detected at 410–508 nm. Fluorescence images were acquired by the PMT/GaAsP PMT detectors.

Images were analyzed using custom MATLAB software developed in our laboratory. The analysis process consists of the segmentation of cells from the background and extraction of the intensity of fluorescence channels (TagRFP, eGFP, LysoTracker) inside cells. First, single cells were segmented. For segmentation, a thresholding-based method was used, where a manually selected threshold was applied. Segmentation was applied to the image created as the sum of all fluorescence channels to achieve segmentation independent of the intensity of individual channels. To achieve better segmentation without noisy pixels, fluorescence images were preprocessed with median filter (7 × 7) and Gaussian filter (standard deviation 1); additionally, binary segmentation was post-processed with morphological closing and removal of small binary connected components (< 5000px). For intensity measurements, the mean value of regions corresponding to cells was extracted from each field of view. Besides individual fluorescence channels, the mean colocalization of TagRFP and GFP was calculated with a pixel-wise multiplication of TagRFP and eGFP channels. Confocal images (autophagy dual labelling) used for the analyses are available online in the Zenodo repository (https://doi.org/10.5281/zenodo.7116549, https://doi.org/10.5281/zenodo.7116549).

### Cell lysis and immunoblot analyses

For protein extraction from cells, these were washed twice with ice-cold 1xPBS and lysed for 20 min on ice in RIPA (Sigma, R0278) or Tris–EDTA SDS lysis buffer (pH 8, 1 M Tris–HCl, 0,5 M EDTA, 10%SDS), supplemented with protease (Roche, 04693132001) and phosphatase inhibitors (MedChemExpress, HY-K0021). Protein concentration was determined using the Pierce BCA protein assay (Thermo Scientific, 23225). Equal amounts of total protein (15 µg) were separated by SDS–PAGE, transferred to a PVDF (GE, GE10600023) or nitrocellulose (Bio-Rad, 1620112) membrane and analysed by using the following antibodies (in a dilution range of 1:500 – 1:5000): phospho 4-EBP1 (Thr 37/46) (#9459); phospho S6K (Thr 389) (#9206), LAMP2 (D5C2P) (#49067) (Cell Signaling), ULK1 (D8H5) (#8054), Atg13 (D4P1K) (#13273), FIP200 (D10D11) (#12436), Atg101 (E1Z4W) (#13492), Phospho-ULK1 (Ser757) (D7O6U) (#14202), Phospho-ULK1 (Ser556) (D1H4) (#5869) from Cell Signaling; CD63 (ab59479), CD9 (ab236630), CD81 (ab109201), Atg7 (ab133528), Atg5 (ab108327), SQSTM1/p62 (ab56416), LC3I/II (ab192890), GABARAP/GABARPL1/GABARAPL2 (ab109364), ACTB (ab16039), Tubulin (ab7219), EpCAM (ab71916) from Abcam; PI3K p100 (sc-365404), BECN1 (E8) (sc-48341), p-mTOR (Ser2448) (sc-293133) LAMP2 (sc-18822), Atg7 (sc-376212), Atg5 (sc-133158), mTOR (sc-517464) from Santa Cruz. Secondary antibodies (diluted 1:1000): HRP-linked Anti-rabbit IgG (#7074) from Cell Signaling, HRP-linked Anti-Rabbit and Anti-Mouse IgG (H + L) from Promega (W4011 and W402B). For detection, the Clarity Western-ECL substrate (Bio-Rad) was used. Blot luminiscence was measured using Azure c400 Imager (Azure Biosystems) and subsequent densitometry was performed in ImageJ gel analyser tools.

For protein extraction from isolated extracellular vesicles, the total yields from all methods used for EVs isolation (including magnetic beads) were lysed in RIPA buffer on ice for 20 min. Concentrations were measured using the Micro BCA™ Protein Assay Kit (Thermo Scientific, 23,235). Proteins were separated by PAGE under nonreducing conditions and transferred to a PVDF membrane (GE, GE10600023) and processed as described above.

### LC–MS

The EVs samples were diluted using SDT buffer (4% SDS, 0.1 M DTT, 0.1 M Tris/HCl, pH 7.6; SDT buffer:EVs ratio 1:1) and incubated in a thermomixer (Eppendorf ThermoMixer® C, 20 min, 95 °C, 1000 rpm). After that, samples were centrifuged (15 min, 20,000 × g) and the supernatant was further processed using filter-aided sample preparation (FASP, 30 kDa cut-off cartridges) as described elsewhere [29] using 0.5 μg of trypsin (sequencing grade; Promega). The resulting peptides were cleared from any residual SDS using liquid–liquid extraction step using ethyl acetate [30] and transferred into the LC–MS vial. The resulting peptides were analysed by nanoElute system (Bruker) connected to timsTOF Pro spectrometer (Bruker) using 68 min long non-linear gradient and diaPASEF acquisition mode. DiaPASEF data were processed in DIA-NN (version 1.8 [31]) in library-free mode against the modified cRAP database (based on http://www.thegpm.org/crap/) and UniProtKB protein database for *Homo sapiens* (https://ftp.uniprot.org/pub/databases/uniprot/current_release/knowledgebase/reference_proteomes/Eukaryota/UP000005640/UP000005640_9606.fasta.gz; version 2021/06, number of protein sequences: 20,600). Protein MaxLFQ intensities reported in the DIA-NN main report file were statistically evaluated. The mass spectrometry proteomics data have been deposited to the ProteomeXchange Consortium via the PRIDE [32] partner repository with the dataset identifier PXD037164. Detailed version of the LC–MS methodology is in Additional file 1.

### EV-TRACK

We have submitted all relevant data of our experiments to the EV-TRACK knowledgebase (EV-TRACK ID: EV220362) [33].

### Real time cell metabolic analysis

To examine the effect of the autophagy modulators or EVs on the cellular metabolism, cells were seeded at the density of 1 × 10^4^ cells per well in a Seahorse 24-well plate (Agilent Technologies, Santa Clara, USA) and incubated for 24 h at 37 °C in the CAF-derived conditioned media. A day before the analysis, Seahorse cartridge (Agilent Technologies) was hydrated and incubated at 37 °C without CO_2_. On the day of the experiment, the Seahorse XFe24 analyzer (Agilent Technologies, Santa Clara, CA, USA) was calibrated using hydrated cartridge. The culture medium was changed to Seahorse XF RPMI medium, pH 7.4 (Agilent Technologies). After incubating cells for 1 h at 37 °C without CO_2_, the Seahorse ATP rate assay was performed by measuring the extracellular acidification rates (ECAR) as a proxy for glycolytic readouts and the oxygen consumption rates (OCR) as a proxy for oxidative phosphorylation readouts. The data were acquired and analysed using Wave 2.6.3 software (Agilent Technologies).

### Statistical analysis

The raw data from proteomic analysis were normalized (log2 normalization) and the quality of each sample was determined. Of all 5405 identified proteins only 2538 had enough proteotypic peptides determined. Outlying replicates were removed and missing values in individual cases were imputed by random distribution around the minimal value of the whole dataset. Moderated t-test was performed in R using LIMMA package, and p values were adjusted on multiple hypothesis testing using Benjamini & Hochberg method [34]. All samples were compared to the control (CTR). The results were visualised using “volcano plots” (EnhancedVolcano package; https://github.com/kevinblighe/EnhancedVolcano). Over-representation analysis (ORA) using Gene Ontology (GO) database was performed using the clusterProfiler 4.0 package from Bioconductor [35]. The individual samples were compared with compareCluster. In the dot-plot visualisation are showed enhanced terms from the cellular compartment and biological process ontologies. Samples were also characterised using Venn diagrams (nVennR package) [36], treemaps (treemap package) [37], and heatmaps (heatmap package) [38]. For data analysis, common packages like tidyverse, ggplot2, or data.table were used [39, 40].

## Results and discussion

### Effect of studied modulators on mTOR and autophagy machinery in FaDu cells

For modulation of the autophagy machinery, nine modulators were chosen (autophinib (APB), CPD18, EACC, bafilomycin A1 (BAFA1), 3-hydroxychloroquine (HCQ), rapamycin (RAPA), NVP-BEZ235 (BEZ), Torin1, and starvation).

**Fig. 1:**
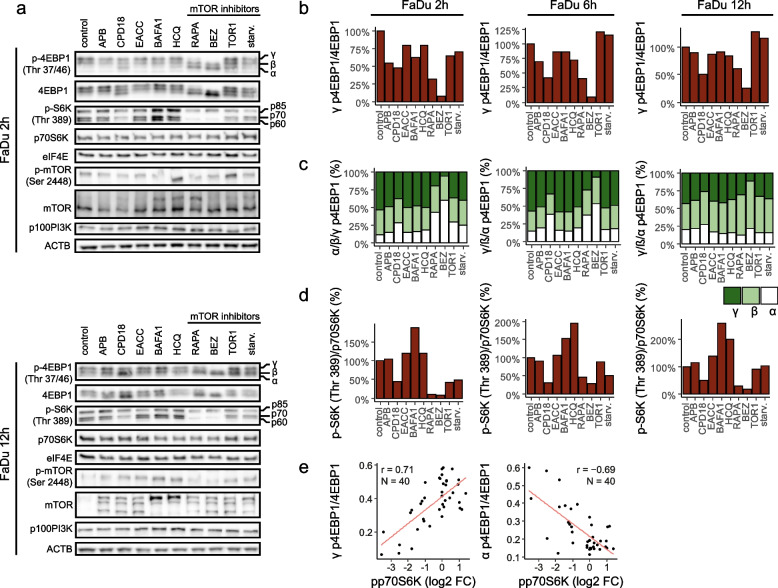
Expression level of proteins in the mTOR/4EBP1 and mTOR/P70S6K signalling pathways. (**a)** Protein expression of p-4EBP1(Thr 37/46), 4EBP1, p-S6K (Thr 389), P70S6K, eIF4E, p-mTOR (Ser 2448), mTOR, p100PI3K; p- indicates phosphorylation. Uncropped western blots for this figure are shown in Additional file 3. (**b)** Densitometric analysis. The ratio of γ 4EBP1 phosphorylation isoform (hyperphosphorylated form) to levels of 4EBP1 protein. (**c)** Densitometric analysis. The levels of α-β-γ 4EBP1 phosphorylation isoforms. (**d)** Densitometric analysis. The ratio of S6K protein phosphorylation on Thr 389 to levels of p70S6K protein. (**e)** Pearson correlation. The level of p-p70S6K was in negative correlation with the presence of α-4EBP1 isoform (hypophosphorylated) (*p* ≤ 0.0001) and in positive correlation with γ-4EBP1 isoform (hyperphosphorylated) (*p* ≤ 0.0001)

As a first step, we evaluated the effect of these autophagy modulators on proteins in the mTOR/4EBP1 and mTOR/S6K signalling pathways (see Fig. 1a, b, c, d, and S2). Treatment of FaDu cells with rapamycin or BEZ prevented the hyperphosphorylation of 4EBP1 (the α-β-γ 4EBP1 isoforms represent the phosphorylation status with α being hypophosphorylated and γ being hyperphosphorylated). While BEZ potently inhibited 4EBP1 hyperphosphorylation throughout the whole duration of treatment (2 h, 6 h, 12 h, 24 h), after rapamycin treatment, 4EBP1 partially recovered in phosphorylation within 12 h despite initial inhibition (2 h). Therefore, cap-dependent translation can be partially maintained in the presence of rapamycin, but probably not in the presence of BEZ. This reemerged 4EBP1 phosphorylation after rapamycin was also shown in Choo et al. study [41]. Interestingly, other mTOR inhibitors Torin1 and starvation showed no such significant effect. The expression of the α isoform was enhanced by CPD18, RAPA, and BEZ.

Rapamycin and BEZ potently inhibited the phosphorylation of p70S6K throughout the duration of treatment (24 h). Phosphorylation of p70S6K was also inhibited by Torin1 and starvation but this inhibition was not so pronounced and long-lasting. The inhibitory effect on p70S6K phosphorylation was observable also after CPD18 treatment. On the other hand, BAFA1 and HCQ enhanced the phosphorylation of all three S6K isoforms (p85S6K, p70S6K, p60S6K). This could be partially explained by higher levels of mTOR after BAFA1 and HCQ treatment compared to control (see Fig. 1a). Consequently, BAFA1 and HCQ can support protein and DNA synthesis [42, 43]. Accordingly, it was shown that lysosomotropic agents can activate the mTORC1 signalling pathway [44] and can induce eIF2α phosphorylation which triggers the integrated stress response [45].

The level of phosphorylation of p70S6K (p-p70S6K) was in negative correlation with the presence of α-4EBP1 isoform (hypophosphorylated) and in positive correlation with γ-4EBP1 isoform (hyperphosphorylated) (see Fig. 1e). This finding is in accordance with the fact that both p-p70S6K and γ-4EBP1 need mTOR to be active.

**Fig. 2:**
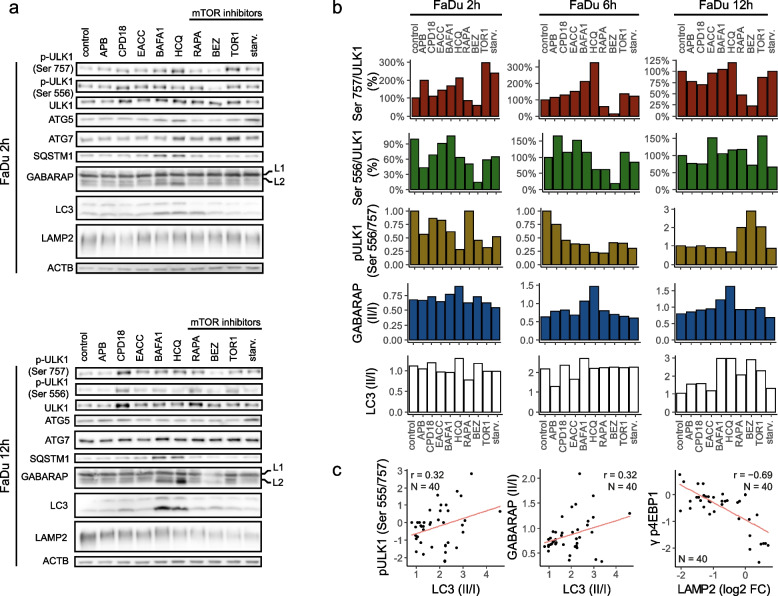
Expression of proteins involved in the autophagy machinery. (**a)** Protein expression of p-ULK1 (Ser 757), p-ULK1 (Ser 556), ULK1, ATG5, ATG7, SQSTM1, GABARAP, LC3, and LAMP2; p- indicates phosphorylation. Uncropped western blots for this figure are shown in Additional file 3. (**b)** Densitometric analyses: the ratio of ULK1 phosphorylation on Ser 757 to ULK1 expression; the ratio of ULK1 phosphorylation on Ser 556 to ULK1 expression; the ratio of ULK1 phosphorylation on Ser 556/ Ser 757; the ratio of GABARAP II/I; the ratio of LC3 II/I. **(c)** Pearson correlation. The ratio of ULK1 phosphorylation on Ser-556 to Ser-757 was in positive correlation with LC3II/I ratio (*p* = 0.046). GABARAPII/I ratio and LC3II/I ratio were in positive correlation (*p* = 0.044). LAMP2 expression was in negative correlation with γ-4EBP1 (*p* ≤ 0.0001)

As a second step, we evaluated the effect of modulators on proteins involved in the autophagy machinery (see Fig. 2a, b, and Additional file 1 Fig. S3). Phosphorylation of ULK1 at Ser-556 stimulates autophagy, whereas phosphorylation at Ser-757 is inhibitory. Treatment with CPD18, EACC, BAFA1, HCQ, RAPA, BEZ, and Torin1 increased ULK1 expression in the time frame 2 h-12 h. The effect of CPD18 was the strongest and long-lasting (2 h, 6 h, 12 h, 24 h). Increased ratio Ser-556/Ser-757 indicating ULK1 activation was clearly observable due to mTOR inhibitors after 12 h (RAPA, BEZ, Torin1). Starvation showed this effect only after 24 h (see Additional file 1 Fig. S3). On the other hand, autophagy inhibitors decreased this ratio after 6 h treatment. No significant changes in Ser-556/Ser-757 ratio between autophagy inhibitors and control were observable at the time point of 12 h. Ratio Ser-556/Ser-757 indicating ULK1 activation was in positive correlation with LC3II/I ratio. GABARAPII/I ratio and LC3II/I ratio were also in positive correlation. LAMP2 expression was in negative correlation with γ-4EBP1 (expressing mTOR activity) (see Fig. 2c). Accordingly, it was shown that activation of mTORC1 by free fatty acids suppresses LAMP2 expression [46]. Starvation led to the accumulation of ATG5. Overexpression of ATG5 in mice was shown to activate autophagy and extend lifespan [47]. P62 (SQSTM1), GABARAP-II, and LC-3II were accumulated after BAFA1 and HCQ treatment during all time points. LC3II/I ratio was decreased after EACC and APB treatment (at 6 h). EACC may increase LC3II degradation by the proteasome [48].

**Fig. 3:**
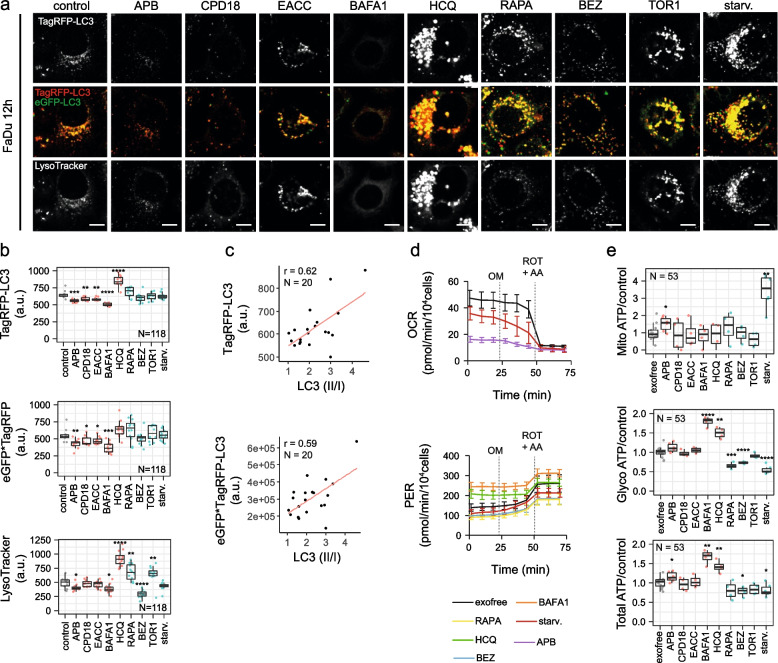
Autophagic flux and energetic consequences of autophagy manipulation. (**a)** Autophagic flux in FaDu cells after 12 h-lasting effect of treatment traced with an mRFP-GFP-LC3 tandem construct; scale bar equals 10 µm. Autophagosomes and autolysosomes are labelled in yellow and TaqRFP, respectively. (**b)** The mean RFP signal (signal of autolysosomes) was significantly diminished after APB, CPD18, EACC, and BAFA1 and enhanced after HCQ treatment. The intensity of the yellow signal (situation where the autophagosome is positive for both red and green signal) was significantly diminished after APB, CPD18, EACC, and BAFA1 treatment indicating decreased autophagic flux. Significantly increased LysoTracker fluorescence intensity was observed for cells treated with HCQ, Torin1, and rapamycin. On the other hand, BAFA1 caused the vanishing of the fluorescent signal of acidic organelles from the cytoplasm. P-values from group comparisons based on the t-test are shown. Asterisks represent statistical significance (* *p* < 0.05; ** *p* < 0.01; *** *p* < 0.001). One dot represents single cell. (**c)** Pearson correlation. LC3II/I ratio assessed by WB was in positive correlation with LC3-mRFP signal (red = autolysosomes) (*p* = 0.0003) and with yellow signal (meanRFP-times-GFP) (*p* = 0.006). (**d)** and** (e)** ATP production from glycolysis and OXPHOS based on Seahorse parameters OCR (oxygen consumption rate) and ECAR (extracellular acidification rate) obtained from XF Real-Time ATP Rate Assay before and after autophagy inhibitors treatment; PER = Proton efflux rate, rate of protons extruded into the extracellular medium during glycolysis. OM = oligomycin; ROT = rotenone; AA = antimycin A. P-values from group comparisons based on the t-test are shown. Asterisks represent statistical significance (* *p* < 0.05; ** *p* < 0.01; *** *p* < 0.001). One dot represents a replicate in a single well

Autophagic flux was also morphologically traced with the mRFP-GFP-LC3 tandem construct (see Fig. 3a, b, Additional file 1 Fig. S4 and S5), where autophagosomes and autolysosomes were labelled in yellow and red, respectively. GFP fluorescence can be quenched by the low pH inside the lysosomes, whereas mRFP fluorescence is stable. Mean yellow (autophagosomes) and red (autolysosomes) signals per cell were detected at the time point of 12 h and 24 h (for a wider field of view see Additional file 1 Fig. S4 and S5). The intensity of the yellow signal (situations where autophagosome is positive for both red and green signal; calculated as a multiple of signals of the green and red channels; further designated as "yellow signal") was significantly diminished after APB, CPD18, EACC, and BAFA1 treatment.Some increase in the yellow signal was observed after RAPA, Torin1, and starvation (see Fig. 3a, b), however, this increase was statistically insignificant. The meanRFP signal was largely diminished after APB, CPD18, EACC, and BAFA1. The simultaneous decrease of both yellow and red signals indicates decreased autophagic flux [49]. The meanRFP signal was enhanced after HCQ indicating swelling of autolysosomes or their increased numbers. Although LC-3II was accumulated after BAFA1 treatment during all time points on WB, the yellow and red signal of the mRFP-GFP-LC3 tandem construct was diminished after BAFA1 treatment. It was found recently that whereas BAFA1 treatment increases the cellular levels of LC3-II, it results in a reduced recruitment of LC3 to autophagosomes. In contrast, HCQ, which increases the content of LC3-II in a similar fashion to BAFA1, did not affect the autophagosomal recruitment of LC3 [48].

LysoTracker-based fluorescent staining specific for lysosomal structures and other acidic organelles (autolysosomes) was applied to analyze the effect of autophagy modulators on lysosomes. Increased LysoTracker fluorescence intensity was observed within cells treated with HCQ (see Fig. 3a, b) indicating an increase in lysosomal size (probably lysosomal swelling) and in the number of lysosomes. HCQ can diffuse across the membranes of cells and organelles to acidic cytoplasmic vesicles (late endosomes, autolysosomes, and lysosomes). Once protonated, HCQ is trapped in the acidic organelles and can no longer freely diffuse out. This effect can cause lysosomal swelling. Lysosomotropic agents have also been shown to activate master lysosomal biogenesis transcription factor EB (TFEB) and ultimately lysosomal biogenesis [50]. An increase in lysosomal biogenesis and LysoTracker fluorescence intensity can be also expected after treatment with TFEB-activating agent Torin1 [50] and Akt-inhibiting rapamycin. Rapamycin inhibits Akt and ERK phosphorylation mainly via the mTORC2 signalling pathway because of the silencing of Rictor [51]. Consequently, TFEB activation via Akt inhibition promotes lysosomal biogenesis [52]. An increase in LysoTracker fluorescence intensity after Torin1 and rapamycin was observable after 12 h-treatment but disappeared after 24 h (see Additional file 1 Fig. S5). On the other hand, BAFA1 caused the vanishing of the fluorescent signal of acidic organelles from the cytoplasm due to the blockage of V-ATPase-based acidification. LC3II/I ratio assessed by WB was in positive correlation with the LC3-mRFP signal (red = autolysosomes) and with the yellow signal; see Fig. 3c.

Our results confirmed that the effect of all used autophagy modulators is realized within the first 24 h after the treatment. Therefore, we decided to isolate exosomes after 24 h-lasting treatment.

Real-time cell metabolic analysis revealed that total ATP production is enhanced after APB, BAFA1, and HCQ treatment. On the other hand, BEZ and starvation decreased total ATP production. BAFA1 and HCQ caused an increase of proton efflux rate following oligomycin treatment, indicating an increase of glycolysis; see Fig. 3d and e. Increased glycolysis rates after HCQ was also observed in metastatic renal cancer cells [53]. Lysosomotropic agents can activate the mTORC1 signalling pathway [44] which can upregulate glucose uptake and glycolysis [54]. RAPA, BEZ, and starvation decreased glycolytic production of ATP. As mTORC1 upregulates glucose uptake and glycolysis, inhibition of glycolysis after RAPA, BEZ, and starvation (all can inhibit mTORC1 signalling) could be expected [54]. Inhibition of glycolysis after Torin1 was visible but not statistically significant. Oxidative phosphorylation (OXPHOS) was significantly enhanced by starvation, as evidenced by a decrease in oxygen consumption rate (OCR) by oligomycin treatment.

### Characterization of isolated EVs

**Fig. 4:**
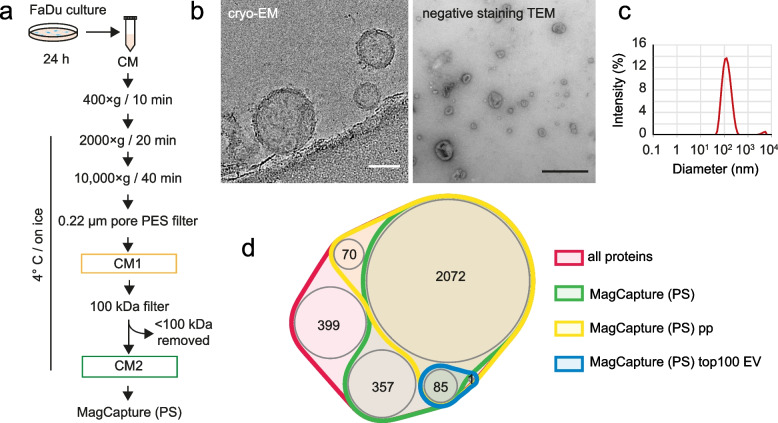
Isolation and characterization of PS-positive EVs. (**a)** Flowchart of the PS-EVs isolation process. (**b)** Cryo-electron microscopy of non-collapsed extracellular vesicles (left), demonstrating the native character of isolated vesicles, with an apparently intact double membrane. The EVs cryo-TEM micrographs were collected on Falcon3 direct electron detection camera at the 120,000 × nominal magnification with the under focus in the range 3–10 µm and the overall dose of < 20 e/Å2; scale bar equals 50 nm. Negative staining transmission electron micrograph of cup-shaped extracellular vesicles (right). Shown is a heterogeneous population of vesicles consisting of a range of sizes (30–200 nm) with low densities typical for exosomes. The extracellular vesicles were isolated by MagCapture isolation kit and embedded in a mixture of 2% uranyl-acetate. EVs were examined at 70 kV with a Morgagni 268D transmission electron microscope. Both Cryo-electron and TEM microscopy were performed on a fresh sample of EVs not subjected to freezing temperatures; scale bar equals 500 nm. (**c)** Size of isolated extracellular vesicles determined by DLS (Dynamic Light Scattering). (**d)** MagCapture (PS) pp = PS-EVs proteins characterized with a sufficient number of proteotypic peptides; MagCapture (PS) topEV = PS-EVs proteins identified in this study and present in the Top 100 proteins list of often identified in EVs according to EV databases ExoCarta and Vesiclepedia

We have isolated small PS-positive EVs (about 118.5 nm; assessed by DLS) using the MagCapture™ Exosome Isolation Kit PS, which proved to be the most suitable of all tested methods; see Fig. 4 and Additional file 1 Fig. S1. Exosomes are typically 30-150 nm in size, which corresponds to the size of EVs isolated using the MagCapture™ method. PS-EVs content shares similarities with exosome content databases. A total of 2538 proteins were identified in the extracted PS-EVs. This number refers to the total number of proteins with enough proteotypic peptides identified in at least one replicate in all analyzed samples (after all autophagy-modulating treatments). When comparing this EVs content with the human exosome proteome databases (ExoCarta and Vesiclepedia), out of 2538 proteins found in PS-EVs, 2028 were present in the ExoCarta database, which constitutes 79.9% of the total protein content of the extracted EVs. In addition, the extracted PS-EVs shared 2349 common proteins with the Vesiclepedia database (92.5% of PS-EVs total protein content). These results confirm the validity of our PS-EVs isolation approach and make our data comparable with established vesicle proteome research. Furthermore, possible changes in representation of MISEV2018-recommended EV proteins [28] due to treatment with autophagy modulators were verified. No significant changes in these proteins caused by used treatments were observed (see Additional file 1 Fig. S1f). This suggests that modulators of autophagy do not significantly affect the predominant type of isolated PS-EVs. The third group of MISEV2018 proteins which includes contaminating proteins, often isolated together with EVs, was represented the least. The most abundant contaminant was the APOB protein. Accordingly, it was shown that lipoproteins (predominantly low-density lipoproteins) can be co-purified with EVs [55].

#### Autophagy modulators significantly change the production of important cytokines by FaDu cells but only a small portion of them is secreted through PS-EVs

**Fig. 5:**
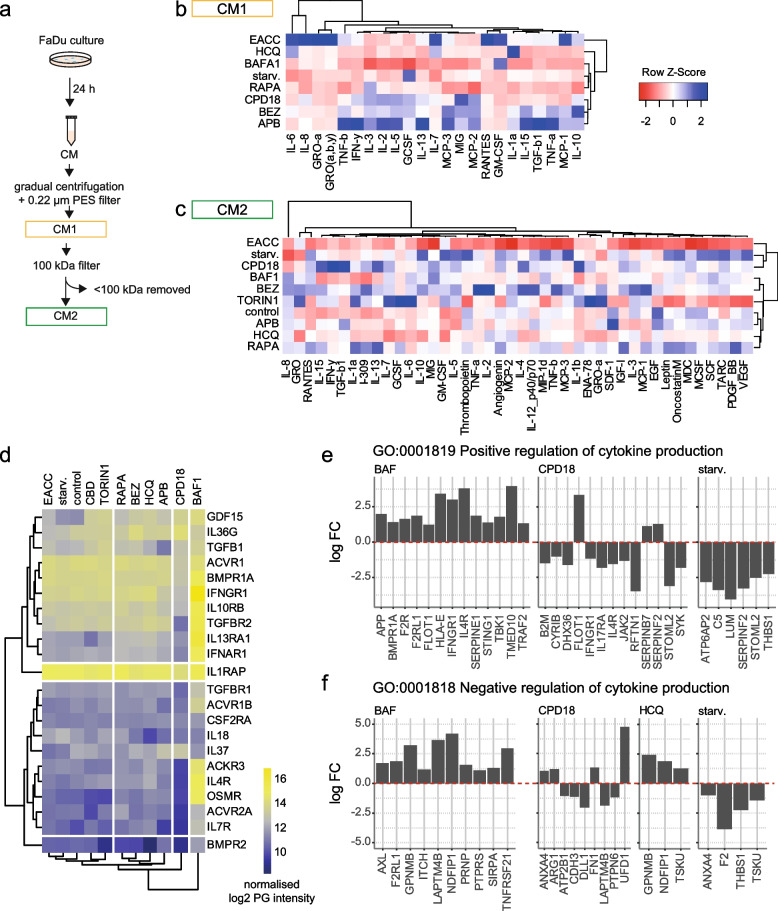
Effect of autophagy modulators on cytokine production by FaDu cells. (**a)** Conditioned media (CM1 and CM2) preparation scheme. In CM1, everything except large EVs (up to 220 nm) and apoptotic bodies can be presented. CM1 involves free cytokines + cytokines in small EVs. CM2 was prepared from CM1. CM1 was concentrated and all molecules smaller than 100 kDa were removed (to remove free cytokines). (**b)** Heatmap of normalized cytokine levels in CM1. IL-6 and IL-8 respond similarly to autophagy modulation. Their expression is most promoted due to autophagy inhibition by EACC and, conversely, starvation suppresses their expression. (**c)** Heatmap of normalized cytokine levels in CM2. The biggest changes were seen in the levels of IL-8. (**d)** Cytokines detected in PS-EVs during proteomic analysis. PG = protein group (**e**) and (**f**) proteins involved in cytokine production were significantly changed in PS-EVs after treatment with autophagy modulators. BAFA1 = bafilomycin, HCQ = 3-hydroxychloroquine, RAPA = rapamycin, BEZ = NVP-BEZ235, starv = starvation, APB = autophinib

In the first part of the experiment, we determined the presence of cytokines in a medium conditioned by FaDu cells that had undergone treatment with different autophagy modulators. The collected conditioned media (CM) were subjected to centrifugation steps to remove cells, debris, and apoptotic bodies. To remove large EVs, the supernatant was centrifuged at 10,000 g for 40 min. To remove any remaining large EVs, a 0.22 μm pore PES filter was used. In the resulting CM1, everything except large EVs (up to 220 nm) and apoptotic bodies can be presented (free cytokines + cytokines in small EVs) (see Fig. 5a).

Modulation of autophagy significantly affected the spectrum of cytokines released by FaDu cells into CM1 (see Fig. 5b). Obviously, the mechanism of action of a given inhibitor is very important as well as its function as an early or late autophagy inhibitor. This finding has implications for the use of autophagy inhibitors as supportive drugs in antineoplastic therapy. For example, the late autophagy inhibitor EACC, which blocks autophagosome-lysosome fusion, significantly increases the production of interleukins) IL-6 and IL-8, which play a critical role in HNSCC carcinogenesis [56]. IL-6 is vitally important for malignant cells. Besides that, it is a critical molecule significantly shaping the tumour microenvironment [57]. The action of EACC also increases the levels of GRO-α, MCP-1, RANTES, and GM-CSF. Both GRO-α and IL-8 can activate TAK1/NFκB signalling via the CXCR2 receptor and are associated with increased cancer aggressiveness [58]. Furthermore, these cytokines can recruit monocytes and macrophages to sites of inflammation, and levels of cytokines were correlated with cancer progression [59–61]. The levels of these factors in CM1 were lower after autophagy induction by starvation or rapamycin and also after FaDu treatment with some early autophagy inhibitors (e.g. CPD18). Cytokine profiles of cancer cells were significantly affected by modulators of autophagy. It can be assumed that this is due, among other things, to changes in the spectrum and composition of small extracellular vesicles produced by FaDu cells. To assess cytokine content in small EVs, CM2 was prepared from CM1. CM1 was concentrated and all molecules smaller than 100 kDa were removed (to remove free cytokines). The changes in cytokine profiles were smaller in this setting. The biggest changes were seen in the levels of IL-8 (see Fig. 5c). Unlike in CM1, changes in IL-6 were insignificant and IL-8 levels were higher after BAFA1 and BEZ treatment due to CM2. EACC did not significantly change the production of IL-6, IL-8, GRO-α, MCP-1, RANTES, or GM-CSF. Thus, we can assume that these cytokines are not released predominantly through small EVs after EACC treatment.

Only one cytokine from the 42 measured cytokines in the cytokine assay was detected in PS-EVs during proteomic analysis, which indicates that secretion of commonly determined cytokines is not predominantly mediated through PS-EVs. This cytokine was transforming growth factor beta-1 proprotein, which is the precursor of the Latency-associated peptide (LAP) and Transforming growth factor beta-1 (TGF-beta-1) chains, which constitute the regulatory and active subunit of TGF-beta-1 (see Fig. 5d).

Nevertheless, other proteins involved in cytokine signalling were present in PS-EVs such as Bone morphogenetic protein receptor type-2, Interleukin-7 receptor subunit alpha, Activin receptor type-2A (ACVR2A), Oncostatin-M-specific receptor subunit beta, Interleukin-4 receptor subunit alpha, Atypical chemokine receptor 3, IL-37, IL-18, Activin receptor type-1B (ACVR1B), TGF-beta receptor type-1 and 2 (TGFBR1, TGFBR2), Interferon alpha/beta receptor 1 (IFNAR1), Interleukin-13 receptor subunit alpha-1 (IL13RA1), Interleukin-10 receptor subunit beta, Interferon-gamma receptor 1, Bone morphogenetic protein receptor type-1A, Activin receptor type-1, IL-36 gamma, and Growth/differentiation factor 15.

Moreover, proteins involved in cytokine production (GO:0001819, 0001818) were significantly changed in PS-EVs after treatment with autophagy modulators (see Fig. 5e and f). For example, levels of Flotillin 1 (FLOT1) were increased in PS-EVs after BAFA1 or CPD18. High expression of FLOT1 correlates with tumour progression [62]. Levels of Lysosome-associated transmembrane protein 4B (LAPTM4B) were increased in PS-EVs after BAFA1 and decreased after starvation. Elevated LAPTM4B levels contribute to chemotherapy resistance in cancer [63]. Levels of Tsukushi (TSKU), that enable TGF-β binding activity, were increased in PS-EVs after HCQ treatment and decreased after starvation. Levels of SERPIN proteins in PS-EVs were also changed due to treatment with autophagy modulators. Serpins can promote cancer cell survival and vascular cooption in metastasis [64].

#### Autophagy modulators significantly change the protein content of PS-EVs. Many of these proteins are important signalling molecules

**Fig. 6:**
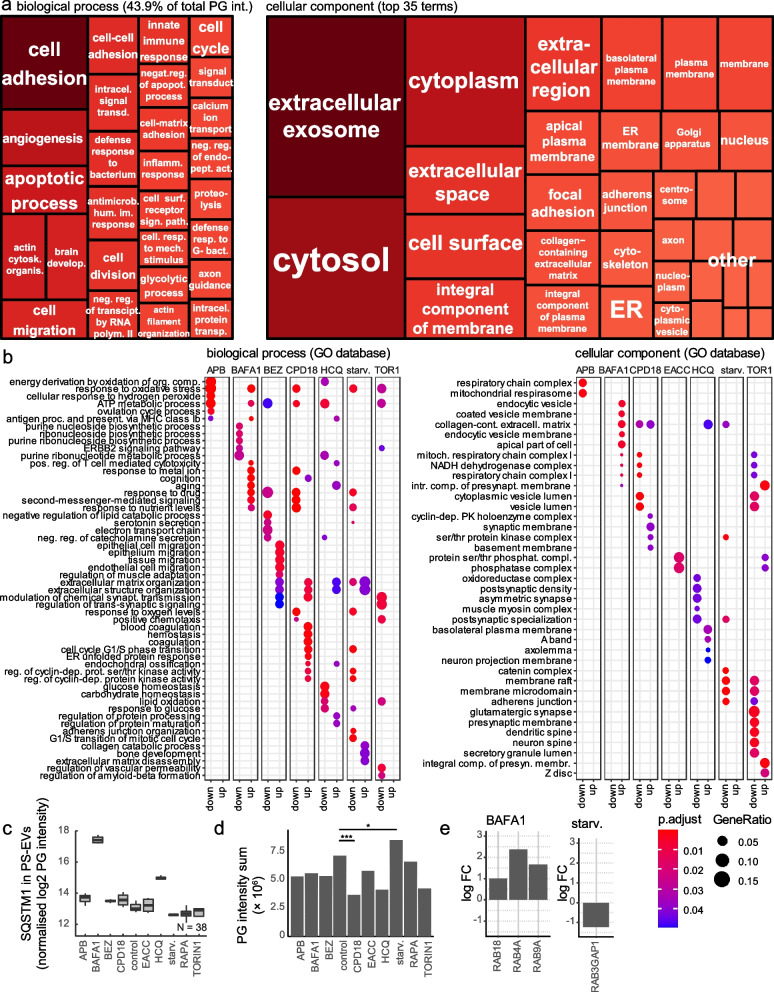
Protein content of PS-EVs. (**a)** Representation of proteins contained in PS-EVs in biological processes and cellular components. The most abundant proteins in PS-EVs were proteins typical for extracellular exosomes, cytosol, cytoplasm, and cell surface involved in cell adhesion, angiogenesis, and apoptotic processes. PG = protein group (**b**) Categories from the GO database. Changes in biological processes and the representation of PS-EVs proteins in particular cellular compartments caused by autophagy modulators. (**c)** Autophagy modulators change the levels of p62/SQSTM1 in PS-EVs. Bafilomycin and HCQ treatment induces the inclusion of p62/SQSTM1 into PS-EVs. PG = protein group (**d**) Autophagy modulators significantly change the representation of mitochondrial proteins in PS-EVs. PG = protein group (**e**) Autophagy modulators significantly change the representation of some Rab proteins in PS-EVs

The most abundant proteins in PS-EVs were proteins typical for extracellular exosomes, cytosol, cytoplasm, and cell surface involved in cell adhesion, angiogenesis, and apoptotic processes; see Fig. 6a and b. The protein content of PS-EVs was the most significantly influenced by HCQ, BAFA1, CPD18, and starvation (see Additional file 2 and Additional file 1 Fig. S6). The numbers of proteins identified after each treatment and in each replicate are shown in Additional file 2.

39 proteins were significantly enriched in PS-EVs after HCQ treatment and 8 were significantly less represented compared to control PS-EVs. 292 proteins were significantly enriched in PS-EVs after BAFA1 treatment and 11 were significantly less represented compared to control PS-EVs. 116 proteins were significantly enriched in PS-EVs after CPD18 treatment and 179 were significantly less represented compared to control PS-EVs. 10 proteins were significantly enriched in PS-EVs after starvation and 118 were significantly less represented compared to control PS-EVs. Both lysosome-affecting compounds BAFA1 and HCQ induced the inclusion of p62/SQSTM1 into PS-EVs (Fig. 6c**)**. Similar enrichment of SQSTM1 in exosomes after BAFA1 was also seen in Minakaki et al. study [65] and after lysosomal impairment [66]. The extracellular SQSTM1 can bind to membrane insulin receptors to activate glycolysis and the production of pro-inflammatory cytokines [67]. Both lysosome-affecting treatments identically increased the abundance of the same 35 proteins in PS-EVs (for example BRI3 = Brain protein I3, GLG1 = Golgi glycoprotein 1, GRN = Granulin, or TMEM59 = Transmembrane protein 59). These proteins are involved in biological processes such as vacuolar transport (GO:0007034) or lysosomal transport (GO:0007041). BRI3 plays an important role in TNF-induced cell death, can be localized to lysosomes, and may function through lysosomes. The antisense RNA against BRI3 promoted resistance to TNF-induced cell death [68]. GLG1 (alias Cysteine-rich fibroblast growth factor receptor) was shown to be a potentially useful auxiliary marker for immunohistochemical diagnosis of Ewing sarcoma and decoy receptor for FGF [69, 70]. As FGFR1 (Fibroblast growth factor receptor 1) activation can suppress autophagy [71], GLG1 presence can support the induction of autophagy. Granulin (GRN) is a potent mitogen and growth factor implicated in many human cancers [72]. TMEM59 acts as a regulator of autophagy by promoting the lipidation of LC3 (MAP1LC3A, MAP1LC3B or MAP1LC3C) and subsequent activation of autophagy [73].

BAFA1 also enhanced the inclusion of autophagy regulators GABARAPL2 (enhanced also by HCQ) and LAMP2, and transporter SLC38A2 into PS-EVs. Increased expression of glutamine transporter SLC38A2 promotes glutamine dependence and oxidative stress resistance and is associated with a worse prognosis in triple-negative breast cancer [74]. According to the GO analysis (see Fig. 6b), proteins, whose exosomal abundance is affected by BAFA1 or HCQ, are predominantly part of the ATP metabolic processes = GO:0046034 (BAFA1-upregulated, HCQ-downregulated), purine nucleoside biosynthetic process = GO:0042451 (BAFA1-downregulated, HCQ-downregulated), and aging = GO:0007568 ((BAFA1-upregulated, HCQ-upregulated). The predominant cellular compartments in which the detected proteins occur inside the cell are shown in Fig. 6b. Components of the mitochondrial respiratory chain complex 1 are enriched in the PS-EVs after BAFA1 treatment.

CPD18 increased the abundance of MYCBP (MYC binding protein), OIT3 (Oncoprotein-induced transcript 3 protein), AFP (α-fetoprotein), GCHFR (GTP cyclohydrolase 1 feedback regulatory protein), or CLEC3B (C-type lectin domain family 3 member B) in PS-EVs. Overexpression of MYCBP can promote invasion and migration in gastric cancer [75]. This protein was also more abundant in PS-EVs after HCQ and BAFA1 treatment. OIT3 mediates macrophage polarization and facilitates hepatocellular carcinoma progression [76]. AFP can promote cancer progression by suppressing the HuR-mediated Fas/FADD apoptotic pathway [77]. Over-expression of GCHFR attenuates LPS and cytokine-stimulated nitric oxide production [78]. Fibroblast collagen synthesis is enhanced by nitric oxide (NO). Fibroblasts can be stimulated by cytokines to synthesize NO, while wound-derived fibroblasts synthesize NO spontaneously [79]. Downregulation of exosomal CLEC3B in hepatocellular carcinoma promoted metastasis and angiogenesis via AMP-activated protein kinase (AMPK) and Vascular endothelial growth factor (VEGF) signals [80]. On the other hand, the abundance of Myosin X (MYO10), Bcl-2-like protein 12 (BCL2L12), or the amount of many important proteins regulating mTOR, Wnt, or cytokine and growth factor signalling was decreased after CPD18 treatment in PS-EVs (e.g.: TNFAIP = TNF-alpha-induced protein 3, TNFAIP8L1 = TNF-alpha-induced protein 8-like 1, MEAK7 = mTOR-associated protein, Eak-7 homolog, TGFBR2 = TGF-β receptor type 2, TNIK = TRAF2 And NCK Interacting Kinase, CCNY = Cyclin-Y, PLK1 = Polo-like kinase 1, IL4R = Interleukin-4 receptor, COPZ1 (Coatomer protein complex subunit zeta 1), IL17RA = Interleukin 17 receptor A, JAK2 = Janus Kinase 2). MYO10 regulates genome stability, through which it mediates inflammation in cancer. Depletion of MYO10 ameliorated genomic instability and reduced inflammation signalling [81]. BCL2L12 inhibits the p53 tumour suppressor and contributes to intense therapeutic resistance of gliomas [82]. According to the GO analysis (see Fig. 6b), proteins whose exosomal abundance is affected by CPD18 are part of the cell cycle G1/S transition = GO:0044843 (upregulated), ATP metabolic processes = GO:0046034 (downregulated), response to oxidative stress = GO:0006979 (downregulated), response to drugs = GO:0042493 (downregulated), and response to oxygen levels = GO:0070482 (downregulated). The predominant cellular compartment in which the detected proteins occur inside the cell is shown in Fig. 6b. Components of the mitochondrial respiratory chain complex 1 are less presented in the PS-EVs after CPD18 treatment.

Starvation increased the abundance of MMP13 (Matrix metalloproteinase 13), which can promote angiogenesis, and decreased the abundance of ALDH1A1 = Aldehyde dehydrogenase 1A1, POSTN = Periostin, RGN = Regucalcin, or GLG1 in PS-EVs [83]. These proteins play an important role in carcinogenesis. Aldehyde dehydrogenase 1 (ALDH1) has been suggested as a putative cancer stem cell marker in several cancer types [84]. Secreted POSTN promoted cancer stemness in head and neck cancer by activating protein tyrosine kinase 7 [85]. RGN promotes dormancy in cancer cells [86]. The abundance of low-density lipoprotein receptor (LDLR), olfactomedin-like 2A (OLFML2A), Stomatin-like protein 2 (STOML2), and stanniocalcin-1 (STC1) in PS-EVs was decreased after both, starvation or CPD18 treatment. Elevated tumour LDLR expression accelerates LDL cholesterol-mediated breast cancer growth [87]. OLFML2A downregulation inhibited glioma proliferation through suppression of Wnt/β-Catenin signalling pathways [88] STOML2 is up-regulated and acts as an oncogenic protein in multiple cancers [89]. STC1 expression is associated with tumour growth and metastasis in breast cancer [90]. Hypothetically, the reduction of these proteins in EVs could contribute to the positive effects of starvation and/or CPD18 during chemotherapy [91]. According to the GO analysis (see Fig. 6b), proteins whose exosomal abundance is affected by starvation are part of the cell cycle G1/S transition = GO:0000082 (downregulated), response to drugs = GO:0042493 (downregulated), and response to oxygen levels and oxidative stress = GO:0070482 and GO:0006979 (downregulated).

Autophagy modulators also significantly changed the representation of mitochondrial proteins and Rab proteins in PS-EVs (see Fig. 6d and e). The highest abundance of mitochondrial proteins in PS-EVs was observable after starvation and the lowest after CPD18 treatment. The representation of mitochondrial proteins in PS-EVs probably reflects ATP production in mitochondria, as this production was highest in FaDu cells during starvation (see Fig. 3e). Recent observation suggests that EV-mediated transfer of mitochondrial content can alter inflammatory responses of recipient cells [92, 93]. Rab proteins coordinate cellular transport because of the effect on vesicle formation, motility, and tethering with target membranes [94]. Rab18, Rab4a, and Rab9a proteins were significantly more presented in PS-EVs after BAFA1 treatment. Rab18 can promote the growth and aggressivity of cancer cells, possibly through STAT3 signalling and the regulation of mitochondrial functions [95–97]. Rab18 knockdown increased autophagy flux and regulated proteostasis [98]. *RAB4A* gene was shown to be amplified in invasive breast cancer [99]. Overexpression of Rab5a and Rab4a proteins may promote the endosomal recycling of epidermal growth factor receptors, which can contribute to tumour progression [100]. Rab9A can play a tumour-promoting role in cancer cells by Akt/mTOR signalling pathway [101]. On the other hand, RAB3GAP1 protein was significantly less presented in FaDu-derived PS-EVs after starvation. RAB3GAP1 can influence protein aggregation and affects autophagy under basal and rapamycin-induced conditions [102].

#### PS-EVs are phagocytosed into recipient fibroblasts and CM2 containing PS-EVs influence the autophagy flux, p21 expression, and metabolic profiles of fibroblasts

**Fig. 7:**
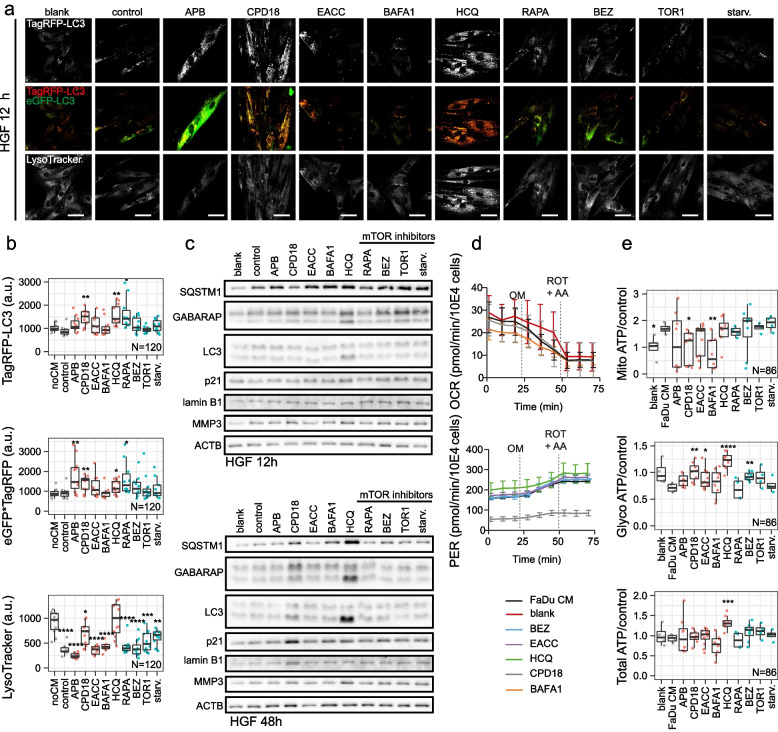
PS-EVs influence the autophagy flux, expression of senescence-associated proteins, and metabolic profiles of fibroblasts. (**a)** Autophagic flux in HGF cells after 12 h-lasting effect of CM2 treatment traced with an mRFP-GFP-LC3 tandem construct. Autophagosomes and autolysosomes are labelled in yellow and red, respectively. LysoTracker-based fluorescent signal is specific for lysosomal structures and acidic organelles. blank = exofree medium incubated at 37 °C 24 h. control = CM2 medium conditioned by non-treated FaDu cells. Scale bar equals 50 µm. Representative fields of view of minimally 11 per treatment. (**b)** CPD18-CM2 medium from FaDu cells pre-treated by CPD18 enhanced autophagy flux in HGF. The intensities of the yellow signal (calculated as meanRFP-times-GFP) and the meanRFP signal from the mRFP-GFP-LC3 tandem construct were significantly enhanced. noCM = HGF without conditioned medium; control = CM2 medium conditioned by non-treated FaDu cells. N indicates number of cells. (**c)** The expression level of proteins involved in the autophagy machinery or senescence after treatment (12 h; 48 h) with CM2 containing PS-EVs. Uncropped western blots for this figure are shown in Additional file 3. (**d)** and (**e**) ATP production from glycolysis and OXPHOS based on Seahorse parameters OCR (oxygen consumption rate) and ECAR (extracellular acidification rate) obtained from XF Real-Time ATP Rate Assay before and after autophagy inhibitors treatment; PER = Proton efflux rate, rate of protons extruded into the extracellular medium during glycolysis. blank = exofree medium incubated at 37 °C 24 h. FaDu CM = CM2 medium conditioned by non-treated FaDu cells. OM = oligomycin; ROT = rotenone; AA = antimycin A. P-values from group comparisons based on the t-test are shown. Asterisks represent statistical significance (* *p* < 0.05; ** *p* < 0.01; *** *p* < 0.001). Each dot represents a measurement in well. N indicates number of cells

To monitor the uptake of isolated PS-EVs by fibroblasts, we stained EVs with PKH67 and then removed the remaining dye using Exosome Spin Columns (MW 3000). The stained EVs were then suspended in 400 µl of medium and added to HGF cell culture growing for 24 h. Image acquisition was performed 24 h after PS-EVs addition. Stained EVs were presented inside fibroblasts (see Additional file 1 Fig. S7a). Autophagic flux in HGF cells after 12 h, 24 h, and 48 h-lasting effects of treatment was traced with an mRFP-GFP-LC3 tandem construct (see Fig. 7a, b, and Additional file 1 Fig. S8). CPD18-CM2 (medium with removed free cytokines; containing small EVs) from FaDu cells pre-treated by CPD18 enhanced autophagy flux in HGF. The intensities of the yellow signal (calculated as meanRFP-times-GFP) and the meanRFP signal from the mRFP-GFP-LC3 tandem construct were significantly enhanced (see Fig. 7a, b, and Additional file 1 Fig. S8). The increase in autophagic flux in HGF after CPD18-CM2 was also observed on WB (enhanced expression of LC3II and GAPARAP after 48 h; see Fig. 7c and S7b. This effect is not the result of residual treatment in CM2, as CPD18 rather decreased the autophagic flux in FaDu cells. On the other hand, the effect of HCQ on fibroblasts is probably caused by residual HCQ in CM2 (persistent high LysoTracker signal), despite the concentrating and following dilution of the concentrated conditioned media to remove the treatment residue. Since, judging by the effect on autophagy, the other treatment residues were successfully removed (no persistent and consistent effect on autophagy), we can speculate that the HCQ residue may be contained in the exosomes.

LysoTracker-based fluorescent signal specific for lysosomal structures and other acidic organelles (autolysosomes) was decreased and the abundance of SQSTM1 protein was increased because of media conditioned with cancer cells (FaDu conditioned medium compared to HGF without CM) (see Fig. 7a and b). Consequently, cancer cells can probably manipulate the lysosomal and autolysosomal numbers in TME fibroblasts, probably through mitochondrial stress [103, 104]. Pretreatment of FaDu cells with CPD18 somewhat reduced this effect of CM2 on LysoTracker signal in HGF. Pretreatment of FaDu cells with CPD18 also led to the accumulation of p21 protein in HGF influenced by FaDu-derived CPD18-CM2 (after 48 h; see Fig. 7c). p21 acts as an inhibitor of the cell cycle by blocking progression through G1/S and belongs among well-established senescence markers [105]. Cellular senescence involves chromatin remodelling and Ivanov et al. showed that an autophagy-lysosome pathway contributes to this [106]. This may explain the simultaneous upregulation of autophagic flux and p21 expression in fibroblasts after CPD18-CM2.

Lamin B1 loss is a senescence-associated biomarker [107]. Nevertheless, no drop in lamin B1 expression was observed after CPD18-CM2 in fibroblasts. It can be caused by LC3–lamin B1 interaction impairing lamin B1 degradation [108]. Treatment of fibroblasts by different kinds of CM2 also significantly influenced the expression of MMP-3 in fibroblasts. MMP-3 is involved in various cancer-associated processes such as angiogenesis, cell growth and cell invasion. Overexpression of MMP-3 was observed in many cancers [109]. The protein expression level of MMP-3 was also upregulated in irradiated senescent cells [110]. CM2 conditioned by FaDu cells, CPD18-CM2, HCQ-CM2, BEZ-CM2, TORIN1-CM2, and starvation-CM2 enhanced the expression of MMP-3 in HGF. All used CM2 caused the accumulation of SQSTM1 in fibroblasts (see Fig. 7c).

Furthermore, Seahorse analyses revealed that the CM2 medium conditioned by FaDu cancer cells increased relative mitochondrial ATP production in HGF. This effect of CM from tumour cells on fibroblasts was previously observed in Kumar et al. study, where HNSCC-secreted bFGF increased mitochondrial oxidative phosphorylation in CAFs [111]. Pretreatment of FaDu cells with CPD18 has again reduced this effect of CM on OXPHOS in fibroblasts. This is in accordance with the lower presence of PS-EVs proteins representing GO pathways related to ATP metabolic processes after CPD18 treatment. A similar effect was also observed after BAFA1 pretreatment. A statistical nonsignificant but visible drop in glycolysis was observed due to CM2 presence. This effect was reduced by CPD18, EACC, or BEZ pretreatment of FaDu cells (see Fig. 7d and e).

## Conclusions

Extracellular vesicles (EVs) are increasingly recognized as key mediators of intercellular communication with prominent roles in many physiological and pathological processes. In this study, we found that autophagy modulators significantly alter the composition of the protein content of phosphatidylserine-positive EVs (PS-EVs) produced by cancer cells. This protein content involves cytokines, mitochondrial proteins, and many important signalling molecules which can actively influence key processes in the tumour microenvironment. Consequently, if autophagy modulators will be used as synergistic agents in cancer therapy, their effect on the protein content of EVs should be considered. The altered protein content of PS-EVs also provides important information about the cellular compartments and processes that are affected by the applied autophagy modulators.

## Supplementary Information

**Additional file 1: Fig. S1.** Isolation and characterization of extracellular vesicles.** Fig. S2.** Expression level of proteins in the mTOR/4EBP1 and mTOR/P70S6K signalling pathways.** Fig. S3.** Expression level of proteins in the autophagy machinery.** Fig. S4. **Autophagic flux in FaDu cells after 12h-lasting effect of treatment traced with an mRFP-GFP-LC3 tandem construct.** Fig. S5. **Autophagic flux in FaDu cells after 24h-lasting effect of treatment traced with the mRFP-GFP-LC3 tandem construct.** Fig. S6.** Volcano plots. Protein content of PS-EVs after treatments which was the most influential (HCQ, BAFA1, CPD18, and starvation). **Fig. S7.** PS-EVs are phagocytosed into recipient fibroblasts and CM2 containing PS-EVs influence the autophagy flux and senescence in HGF.** Fig. S8. **Autophagic flux in HGF cells after 24h and 48h-lasting effect of treatment traced with the mRFP-GFP-LC3 tandem construct.

**Additional file 2: Table S1.** Changes in the representation of PS-EVs proteins caused by autophagy modulators.

**Additional file 3: **Full and uncropped western blots.

## Data Availability

Confocal images (autophagy dual labelling) used for the analyses are available online in the Zenodo repository (https://doi.org/10.5281/zenodo.7116549, https://doi.org/10.5281/zenodo.7116549). The mass spectrometry proteomics data have been deposited to the ProteomeXchange Consortium via the PRIDE [32] partner repository with the dataset identifier PXD037164". We have submitted all relevant data of our experiments to the EV-TRACK knowledgebase (EV-TRACK ID: EV220362) [33].
