# Supplementary material for: Autophagy modulators influence the content of important signalling molecules in PS-positive extracellular vesicles
Source: Cell Commun Signal. 2023 May 24;21:120. doi: 10.1186/s12964-023-01126-z (PMC10210466; doi:10.1186/s12964-023-01126-z)
Supplement: Supplementary file 2 — Additional file 1: Fig. S1. Isolation and characterization of extracellular vesicles. Fig. S2. Expression level of proteins in the mTOR/4EBP1 and mTOR/P70S6K signalling pathways. Fig. S3. Expression level of proteins in the autophagy machinery. Fig. S4. Autophagic flux in FaDu cells after 12h-lasting effect of treatment traced with an mRFP-GFP-LC3 tandem construct. Fig. S5. Autophagic flux in FaDu cells after 24h-lasting effect of treatment traced with the mRFP-GFP-LC3 tandem construct. Fig. S6. Volcano plots. Protein content of PS-EVs after treatments which was the most influential (HCQ, BAFA1, CPD18, and starvation). Fig. S7. PS-EVs are phagocytosed into recipient fibroblasts and CM2 containing PS-EVs influence the autophagy flux and senescence in HGF. Fig. S8. Autophagic flux in HGF cells after 24h and 48h-lasting effect of treatment traced with the mRFP-GFP-LC3 tandem construct. [file 12964_2023_1126_MOESM1_ESM.pdf]

Supplementary material

Autophagy modulators influence the content of important signalling molecules in PS-positive extracellular vesicles

Klara Hanelova[1×], Martina Raudenska[1,2×], Monika Kratochvilova[2], Jiri Navratil[1,2], Tomas Vicar[1,3], Maria Bugajova[1], Jaromir Gumulec[1], Michal Masarik[1,2,4], and Jan Balvan[1*]

[1]Department of Pathological Physiology, Faculty of Medicine, Masaryk University / Kamenice 5, CZ-625 00 Brno, Czech Republic
[2] Department of Physiology, Faculty of Medicine, Masaryk University / Kamenice 5, CZ-625 00 Brno, Czech Republic
[3]Department of Biomedical Engineering, Faculty of Electrical Engineering and Communication, Brno University of Technology, Technicka 3058/10, Brno, Czech Republic
[4]BIOCEV, First Faculty of Medicine, Charles University, Prumyslova 595, CZ-252 50 Vestec, Czech Republic

*Dr. Jan Balvan, Department of Physiology, Faculty of Medicine, Masaryk University, Kamenice 5, CZ-625 00 Brno, Czech Republic; E-mail: jan.balvan@med.muni.cz Phone: +420-5-4949-8526; fax: +420-5-4949-4340
× Klara Hanelova and Martina Raudenska contributed equally to this work.

Supplementary information – Methods

LC-MS

The exosome suspensions were diluted using SDT buffer (4% SDS, 0.1M DTT, 0.1M Tris/HCl, pH 7.6; SDT buffer: exosome ratio 1:1) and incubated in a thermomixer (Eppendorf ThermoMixer® C, 20 min, 95°C, 1000 rpm). After that, samples were centrifuged (15 min, 20,000 x g) and the supernatant used for filter-aided sample preparation (FASP, 30kDa cut-off cartridges) as described elsewhere [24] using 0.5 µg of trypsin during the digestion step (sequencing grade; Promega). The resulting peptides were cleared from any residual SDS using the liquid-liquid extraction step using ethyl acetate [104] and transferred into the LC-MS vial.

LC-MS/MS analyses of all peptides were done using nanoElute system (Bruker Daltonics) connected to timsTOF Pro spectrometer (Bruker). Two columns mode (trap column: µPrecolumn, 300 µm inner diameter, 5mm long; C18 PepMap100, 5µm particles, 100 A, Thermo; separation column: Bruker FORTY, 75µm ID, 400 mm long, 1.9 µm particles, Bruker Daltonics) was used on nanoElute system with default equilibration and modified sample loading conditions (loading volume of 4×sample volume +2 µl loaded at 100 bars). Concentrated peptides were eluted by 68 min long non-linear gradient program (flow rate 300nl/min; gradient started at 3% of mobile phase B, and followed with 30% at 60th min and finally 40% at 68th min of mobile phase B; mobile phase A: 0.1% FA in water; mobile phase B: 0.1% FA in ACN), followed by 12 min wash step at 80% of mobile phase B. The analytical column was placed inside the Column Toaster (40°C; Bruker Daltonics) and connected to the Captive Spray ZDV emitter (part number 1865691, Bruker Daltonics).

MSn data were acquired in data independent acquisition (DIA) mode with base method m/z range of 100-1700 and 1/k0 range of 0.6-1.6 V×s×cm^{-2}. Enclosed DIAparameters.txt file defined m/z 400-1000 precursor range with equal windows size of 20 Th using two steps each PASEF scan and cycle time of 100ms locked to 100% duty cycle.

DiaPASEF data were processed in DIA-NN (version 1.8, https://github.com/vdemichev/DiaNN [26]) in library-free mode against the modified cRAP database (based on http://www.thegpm.org/crap/; 111 sequences in total) and UniProtKB protein database for *Homo sapiens* (ftp://ftp.uniprot.org/pub/databases/uniprot/current_release/knowledgebase/reference_proteomes/Eukaryota/UP000005640/UP000005640_9606.fasta.gz; version 2021/06, number of protein sequences: 20,600). No optional, carbamidomethylation as fixed modification and trypsin/P enzyme with 1 allowed missed cleavages were set during the library preparation. False discovery rate (FDR) control was set to 1% FDR. MS1 and MS2 accuracies as well as scan window parameters were set based on the initial test searches (median value from all samples ascertained parameter values). MBR was switched on.

Protein MaxLFQ intensities reported in the DIA-NN main report file were further processed using the software container environment (https://github.com/OmicsWorkflows), version 4.1.3a. Processing workflow is available upon request. Briefly, it covered: a) removal of low-quality precursors and

contaminant protein groups, b) protein group MaxLFQ intensities log2 transformation, c) filtering out of protein groups not quantified in more than half of the replicates of at least one sample type, d) imputation of the missing values from the random distribution around the global minimal value, e) differential expression analysis using LIMMA statistical test. Proteins with adjusted p-value <0.01 and fold change >2 were considered as potential interacting partners.

The mass spectrometry proteomics data have been deposited to the ProteomeXchange Consortium via the PRIDE [27] partner repository with the dataset identifier PXD037164.

Project Name: Head and neck squamous cell carcinoma extracelular vesicles proteome LC-MS
Project accession: PXD037164
Project URL: https://www.ebi.ac.uk/pride/archive/projects/PXD037164

EV-Track

You may access and check the submission of experimental parameters to the EV-TRACK knowledgebase via the following URL: http://evtrack.org/review.php. Please use the EV-TRACK ID (EV220362) and the last name of the first author (Hanelova) to access our submission.

Supplementary figures

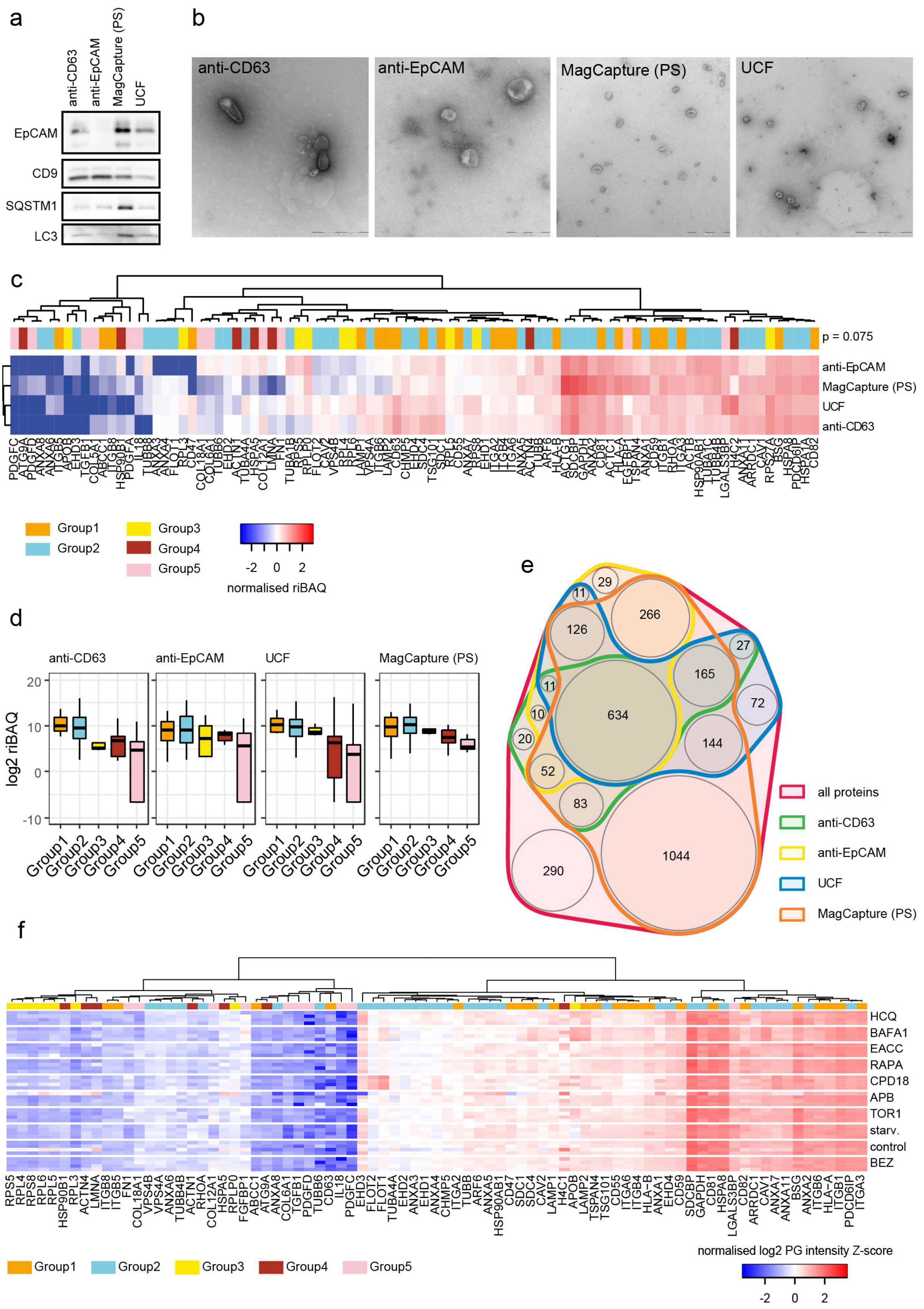

Fig. S1.: Isolation and characterization of extracellular vesicles. ***(a)*** *Protein expression of EpCAM, CD9, SQSTM1, and LC3 in extracellular vesicles obtained by different isolation methods. The amount of total protein was compared within each tetraplet representing the isolation method used. For each method,*

the maximum isolation yield was used. Uncropped western blots for this figure are shown in Additional file 3 **(b)** *Negative-Stain Transmission Electron Microscopy. For anti-CD63 and MagCapture (PS) method 56 000x magnification was used, scale bar equals 500nm; For anti-EpCAM method 140000x magnification was used, scale bar equals 200nm; For UCF method 28000x magnification was used, scale bar equals 1000nm.* **(c)** *Characterization of the MISEV2018 protein content of extracellular vesicles obtained by different isolation methods using liquid chromatography-mass spectrometry (LC-MS).* **(d)** *5 categories of proteins were evaluated to characterize EVs; based on the MISEV2018 recommendations[28]. The fifth category of proteins reflects the native state of isolated exosomes.* **(e)** *The overlap between the protein content of extracellular vesicles obtained by different isolation methods.* **(f)** *Characterization of the MISEV2018 protein content of extracellular vesicles obtained after autophagy modulation.*

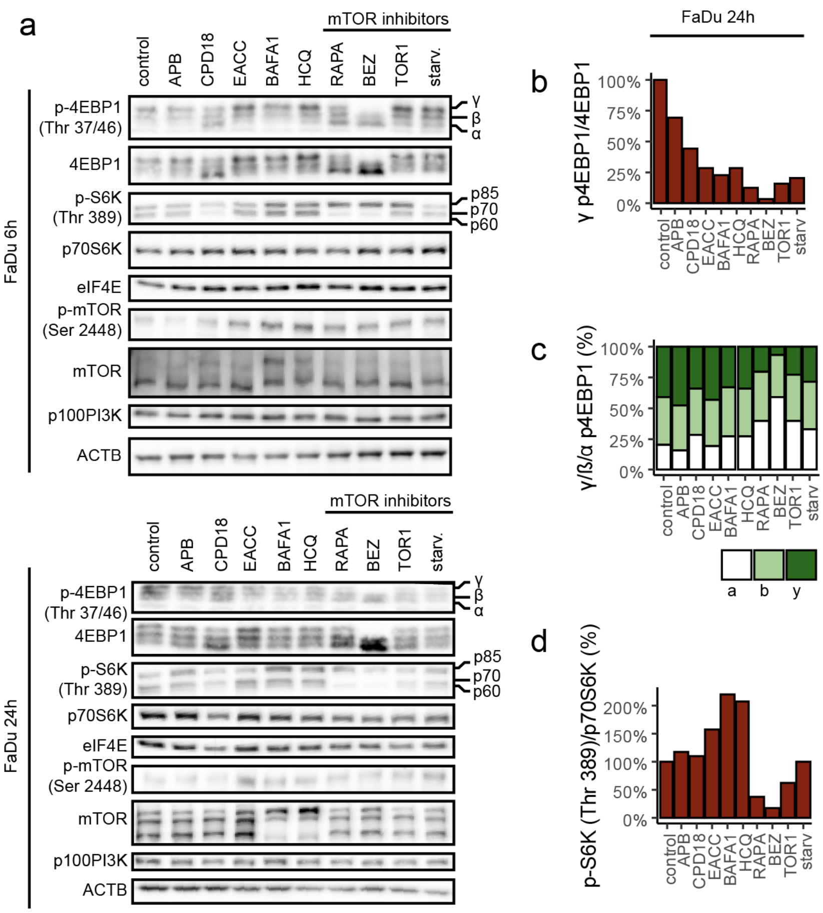

Fig. S2.: Expression level of proteins in the mTOR/4EBP1 and mTOR/P70S6K signalling pathways. ***(a)*** *Protein expression of p-4EBP1(Thr 37/46), 4EBP1, p-S6K (Thr 389), P70S6K, eIF4E, p-mTOR (Ser 2448), mTOR, p100PI3K.* ***(b)*** *The ratio of γ 4EBP1 phosphorylation isoform to levels of 4EBP1 protein; assessed by densitometric analysis.* ***(c)*** *The levels of α-β-γ 4EBP1 phosphorylation isoforms; assessed by densitometric analysis.* ***(d)*** *The ratio of S6K protein phosphorylation on Thr 389 to levels of p70S6K protein; assessed by densitometric analysis. Uncropped western blots for this figure are shown in Additional file 3.*

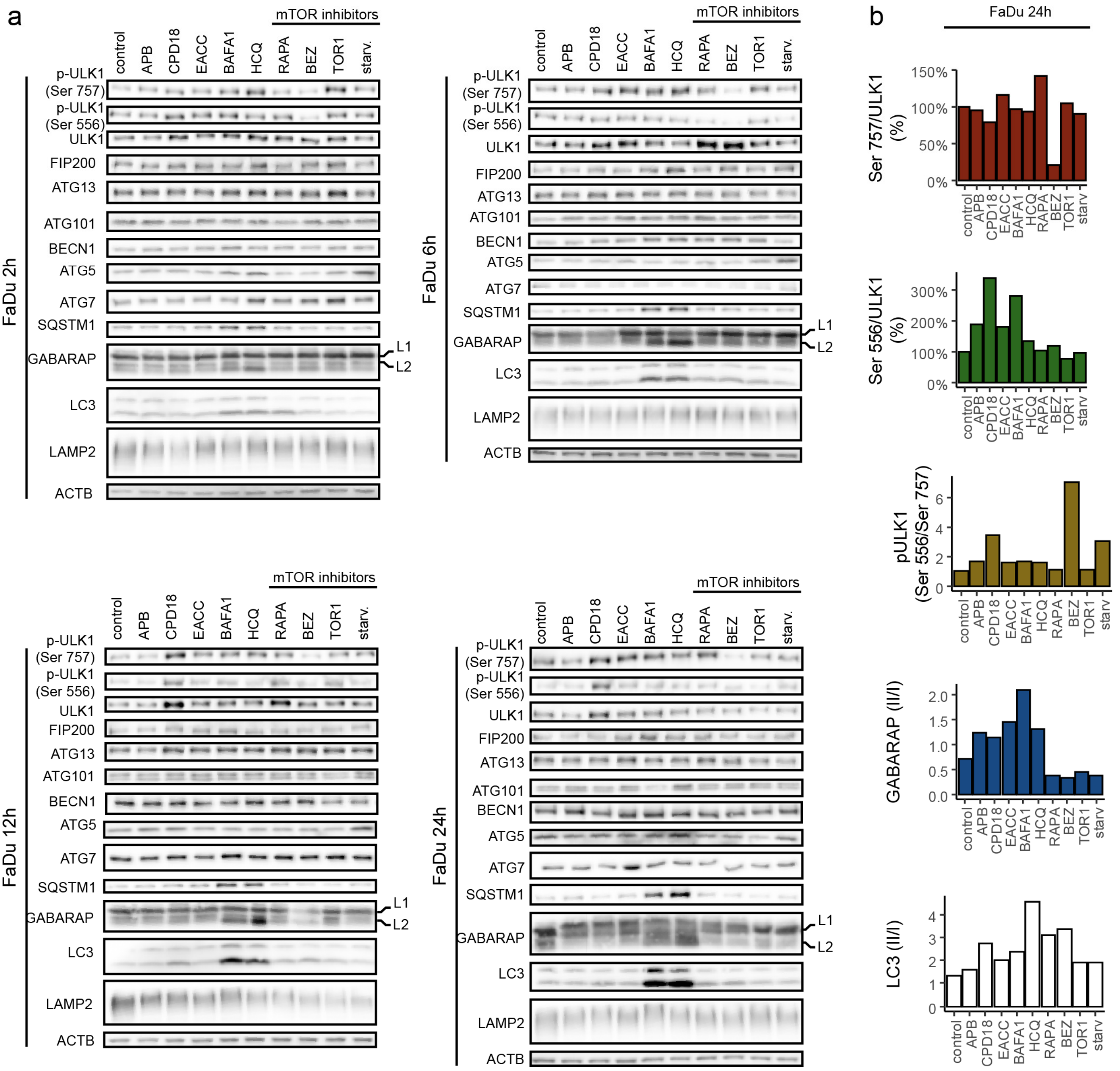

Fig.S3.: Expression level of proteins in the autophagy machinery. (a) *Protein expression of p-ULK1 (Ser 757), p-ULK1 (Ser 556), ULK1, FIP200, ATG13, ATG101, BECN1, ATG5, ATG7, SQSTM1, GABARAP, LC3, and LAMP2.* ***(b)*** *Densitometric analyses: the ratio of ULK1 phosphorylation on Ser 757 to ULK1 expression; the ratio of ULK1 phosphorylation on Ser 556 to ULK1 expression; the ratio of ULK1 phosphorylation on Ser 556 to phosphorylation on Ser 757; the ratio of GABARAP II/I; the ratio of LC3 II/I. Uncropped western blots for this figure are shown in Additional file 3.*

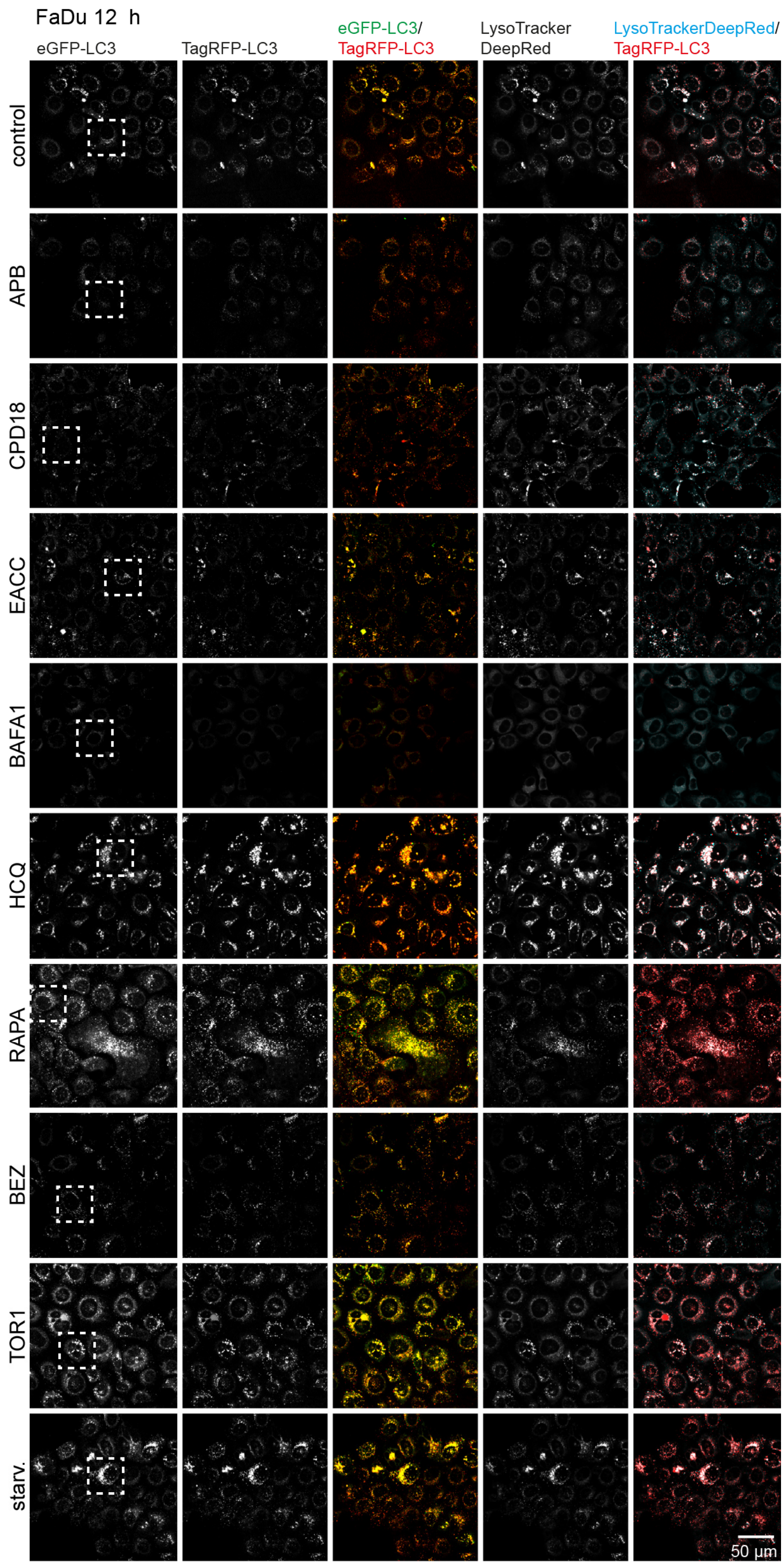

Fig. S4.: Autophagic flux in FaDu cells after 12h-lasting effect of treatment traced with an mRFP-GFP-LC3 tandem construct. *Autophagosomes and autolysosomes are labelled in yellow and red, respectively. LysoTracker-based fluorescent staining specific for lysosomal structures and other acidic organelles (autolysosomes) was applied to analyze the effect of autophagy modulators on lysosomes.*

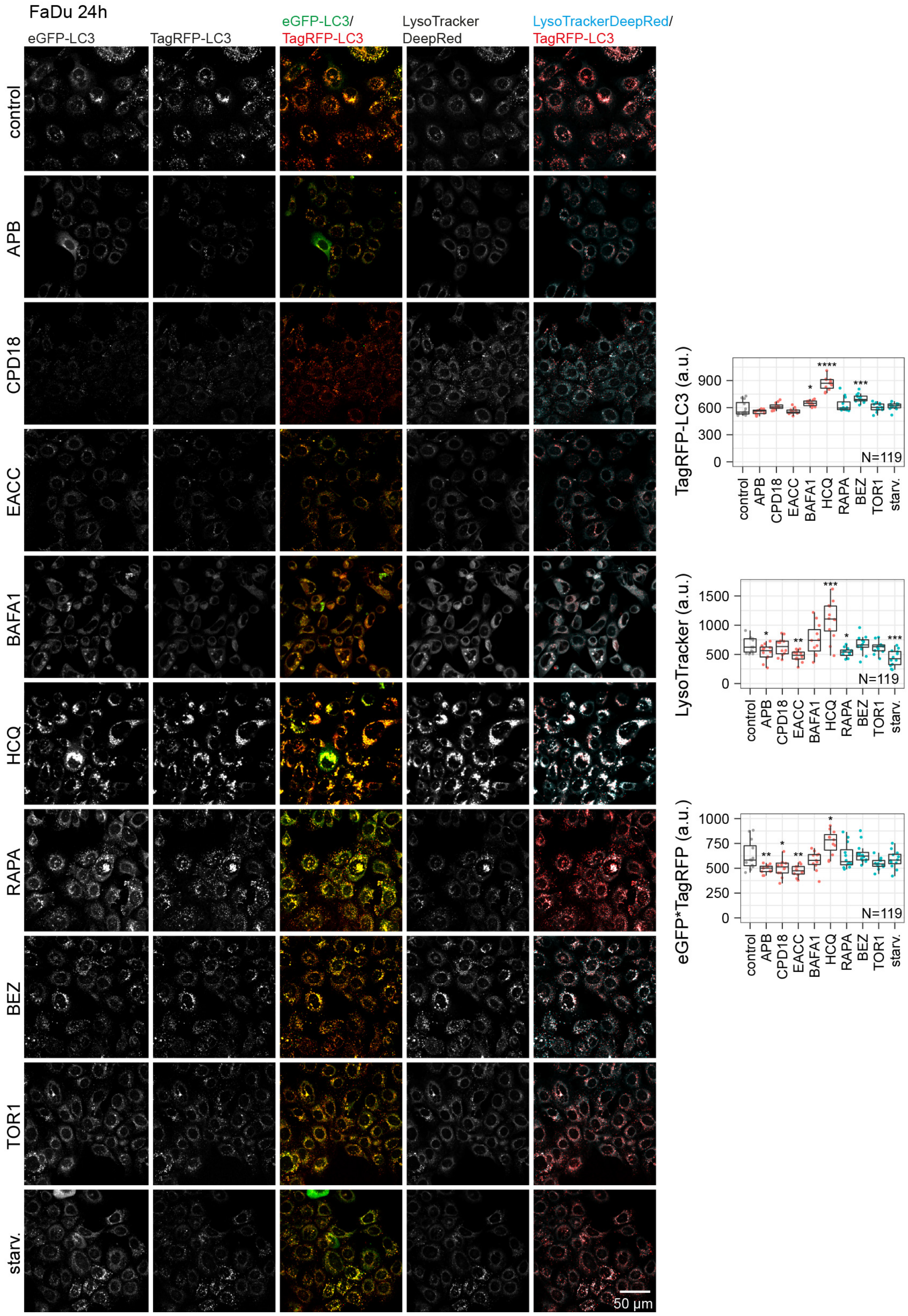

Fig. S5.: Autophagic flux in FaDu cells after 24h-lasting effect of treatment traced with the mRFP-GFP-LC3 tandem construct. *Autophagosomes and autolysosomes are labelled in yellow and red, respectively. The meanRFP signal (signal of autolysosomes) was significantly enhanced after HCQ treatment. The intensity of the yellow signal (situations where the autophagosome is positive for both red and green signal; yellow in merge; calculated as meanRFP-times-GFP) was significantly diminished after APB, CPD18, and EACC, LysoTracker-based fluorescent staining is specific for lysosomal structures and other acidic organelles (autolysosomes). Significantly increased LysoTracker fluorescence intensity was observed for cells treated with HCQ. P-values from group comparisons based on the t-test are shown. Asterisks represent statistical significance (* p<0.05; ** p<0.01; *** p<0.001).*

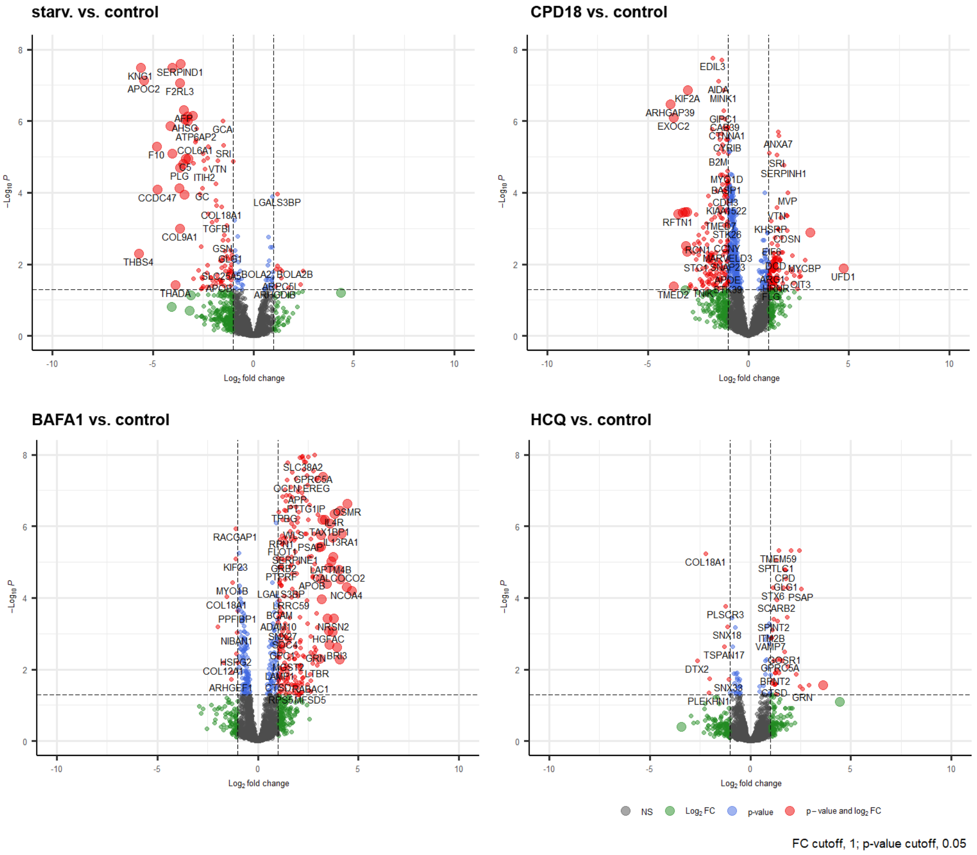

Fig. S6.: Volcano plots. ***Protein content of PS-EVs after treatments which was the most influential (HCQ, BAFA1, CPD18, and starvation).*** *Shown relative to control after a quality check filter.*

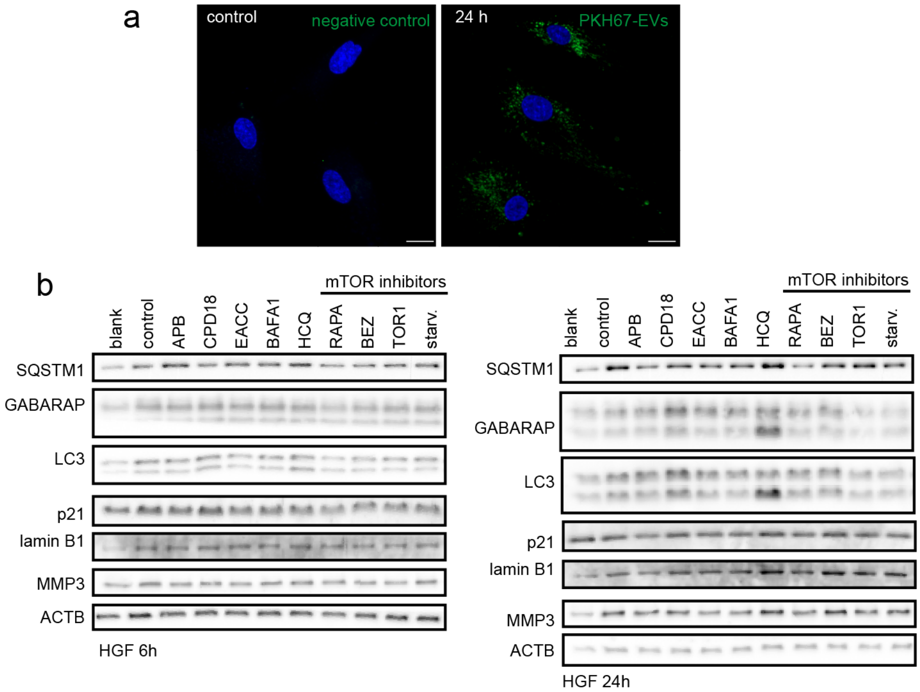

Fig. S7.: PS-EVs are phagocytosed into recipient fibroblasts and CM2 containing PS-EVs influence the autophagy flux and senescence in HGF. ***(a)*** *Fluorescence microscopy; the uptake of isolated PS-EVs by fibroblasts. Image acquisition was performed 24 hours after PS-EVs addition. Control = fibroblasts in exofree medium, scale bar equals 20µm.* ***(b****) The expression level of proteins involved in the autophagy machinery or senescence after treatment (6h; 24h) with CM2 containing PS-EVs. Uncropped western blots for this figure are shown in Additional file 3.*

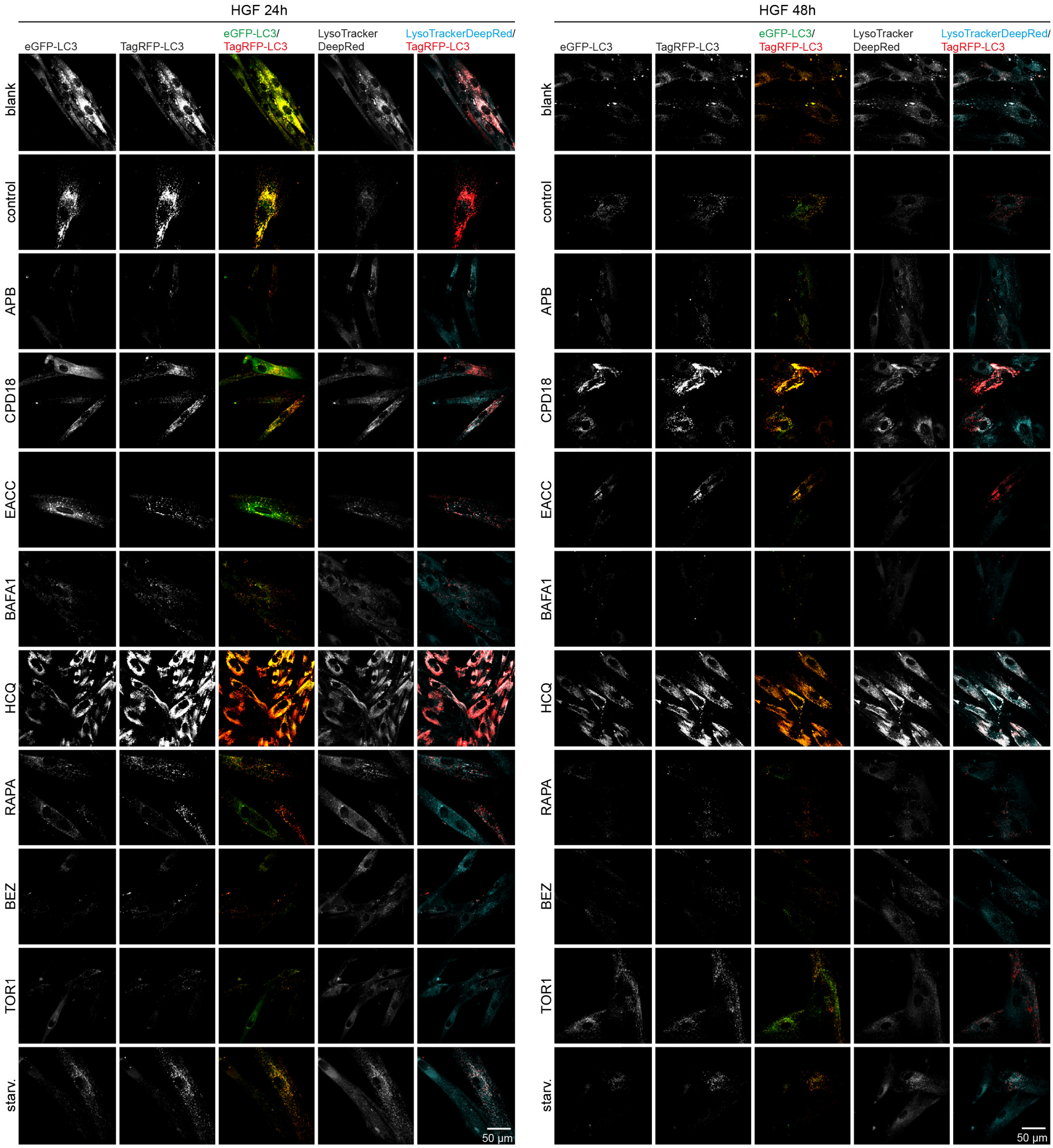

Fig. S8.: Autophagic flux in HGF cells after 24h and 48h-lasting effect of treatment traced with the mRFP-GFP-LC3 tandem construct. *Autophagosomes and autolysosomes are labelled in yellow and red, respectively. LysoTracker-based fluorescent staining is specific for lysosomal structures and other acidic organelles (autolysosomes). blank=exofree medium incubated at 37 °C 24h. control=CM2 medium conditioned by non-treated FaDu cells.*
