# Supplementary material for: Autophagy modulators influence the content of important signalling molecules in PS-positive extracellular vesicles
Source: Cell Commun Signal. 2023 May 24;21:120. doi: 10.1186/s12964-023-01126-z (PMC10210466; doi:10.1186/s12964-023-01126-z)

Full and uncropped western blot for Figure 1 (timepoints 2h and 12h) and Additional File 1 Figure S2 (timepoints 6h and 24h)

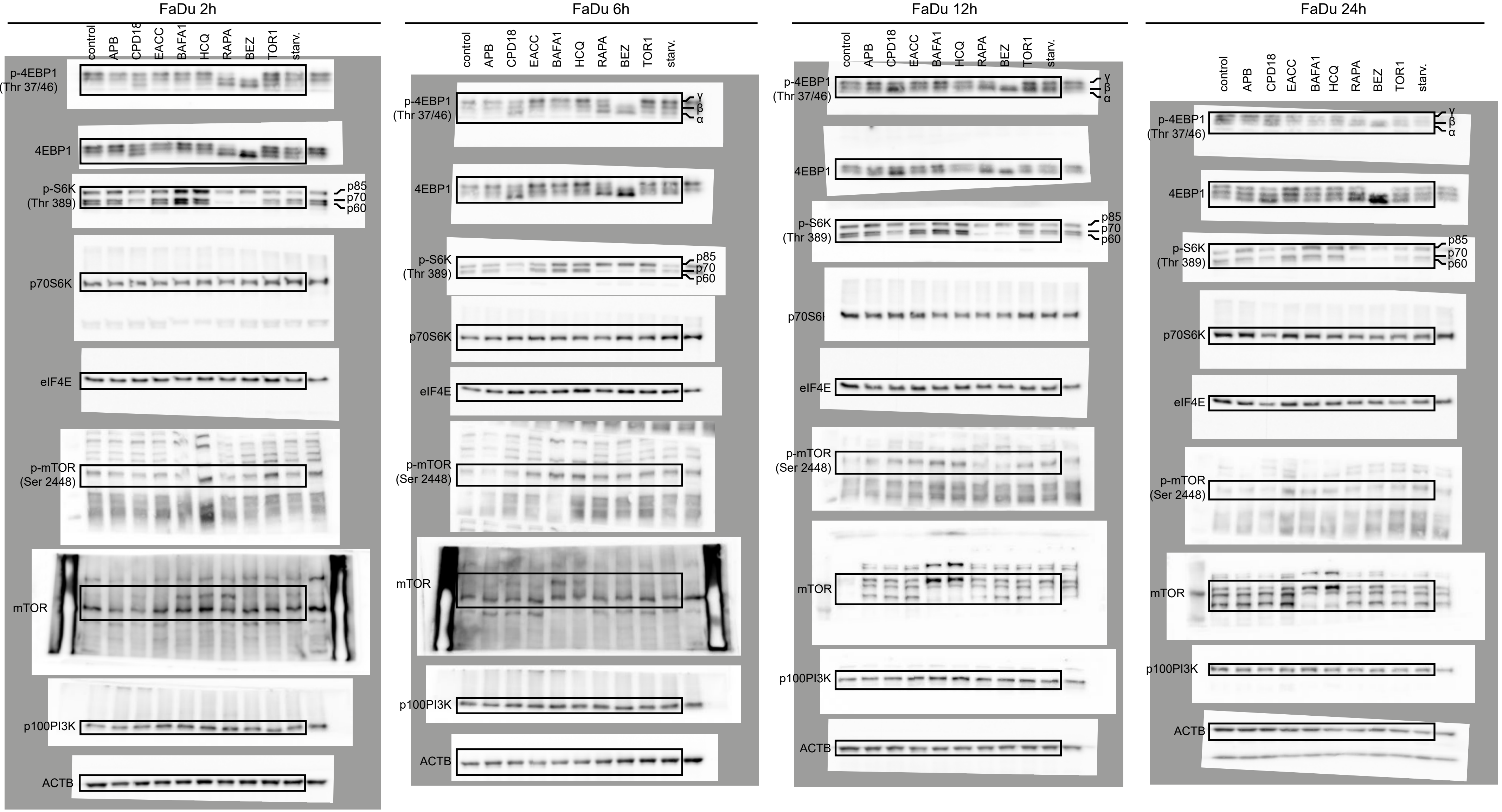

Full and uncropped western blot for Figure 2 (timepoints 2h + 12h, selected blots) and Additional File 1 Figure S3 (remaining blots)

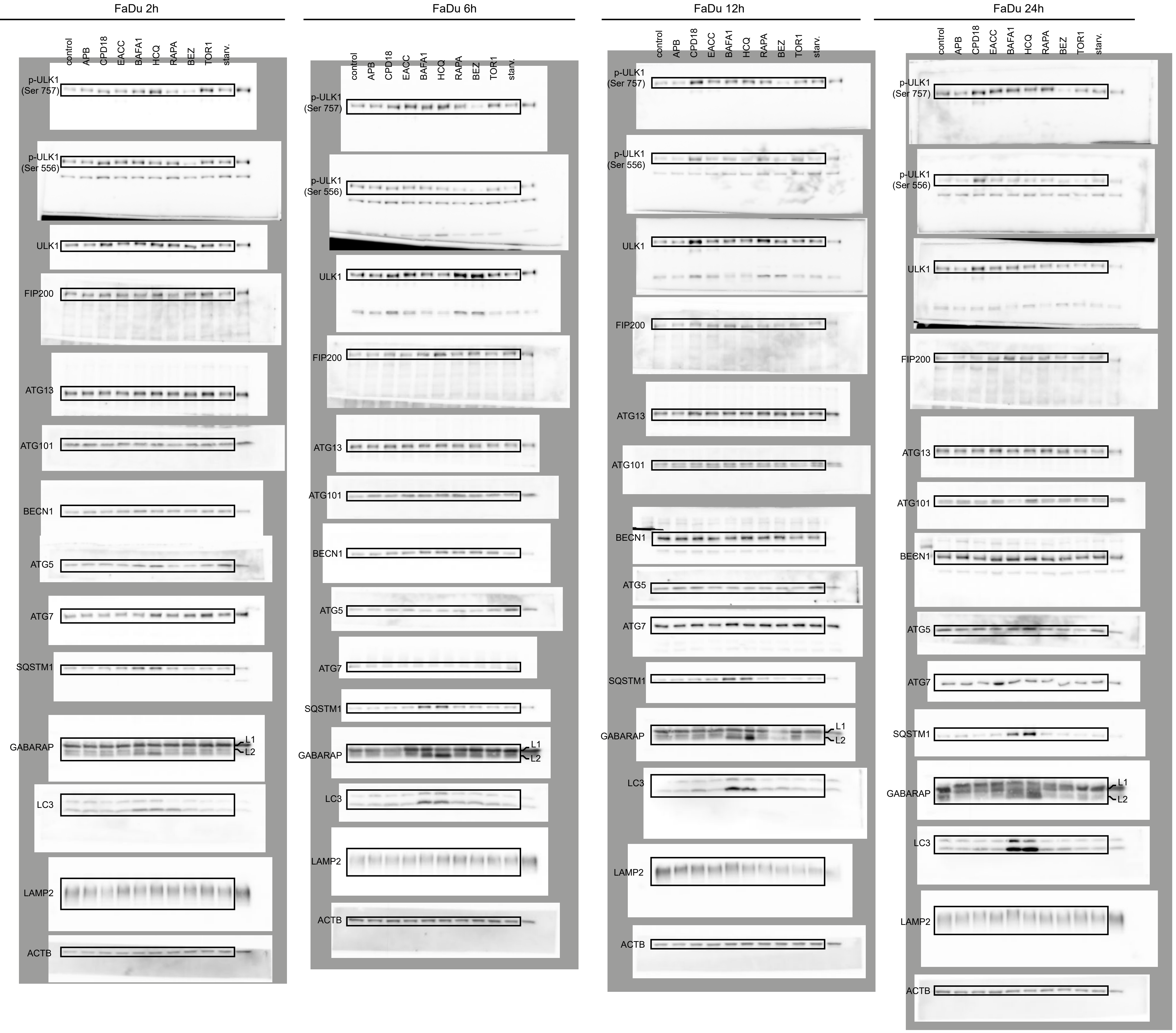

Full and uncropped western blot for Figure 7 (timepoints 12 and 48h) and Additional File 1 Figure S7 (timepoints 6 and 24h)

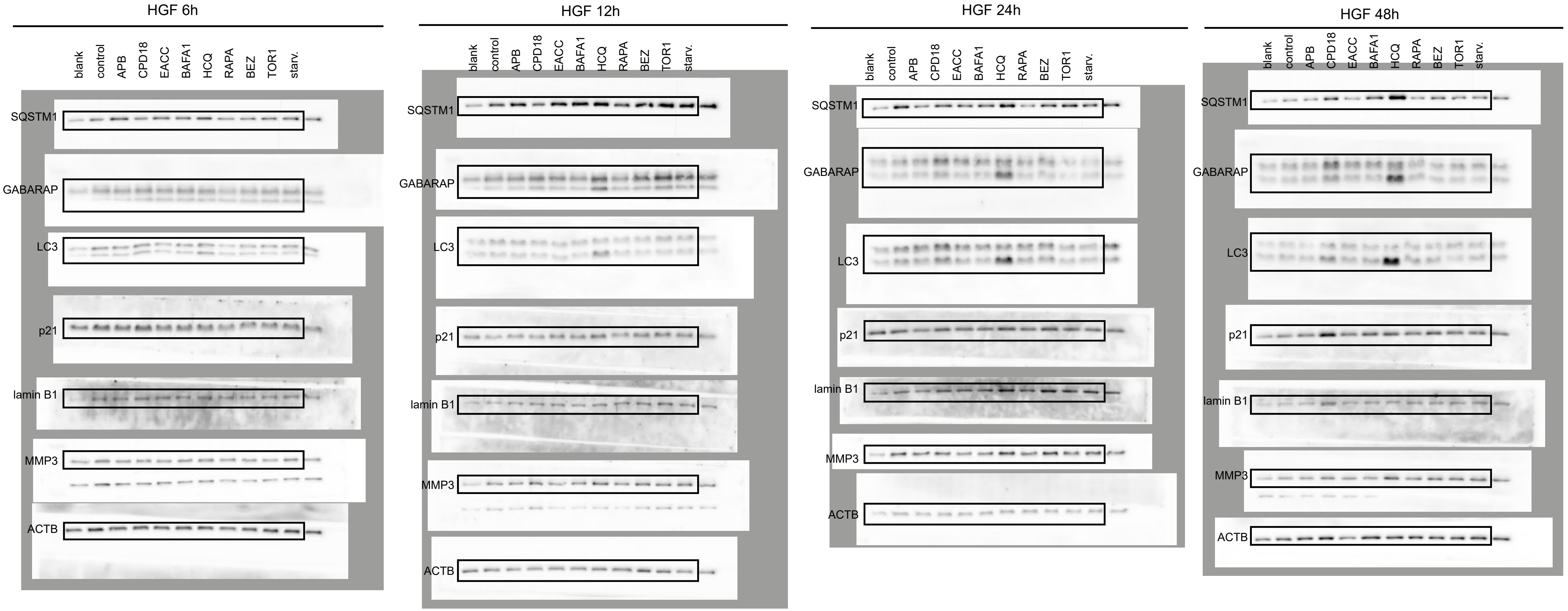

Full and uncropped western blot for Additional File 1 Figure S1

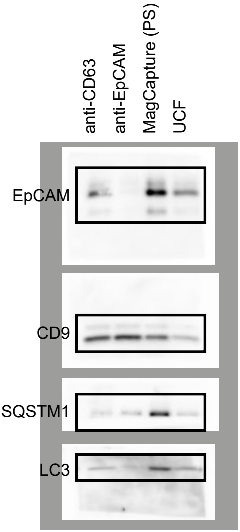

Supplement: Supplementary file 4 — Additional file 3: Full and uncropped western blots. [file 12964_2023_1126_MOESM3_ESM.pdf]
